# Biocoatings with Enhanced Bacterial Viability via Coagulant Dipping and Wet Sintering by Immersion

**DOI:** 10.1021/acsami.6c05652

**Published:** 2026-04-17

**Authors:** Alexia M. J. M. Beale, Kathleen L. Dunbar, Emily M. Brogden, Solomon S. Melides, Richard P. Sear, Stefan A. F. Bon, Suzanne M. Hingley-Wilson, Joseph L. Keddie

**Affiliations:** † School of Mathematics and Physics, 3660 University of Surrey, Guildford, Surrey GU2 7XH, U.K.; ‡ Department of Chemistry, 2707 University of Warwick, Coventry CV4 7AL, U.K.; § Discipline of Microbiology, Infection and Immunity, 3660 University of Surrey, Guildford, Surrey GU2 7XH, U.K.

**Keywords:** latex, film formation, living materials, porosity, bacteria, colloids, coalescence

## Abstract

Biocoatings are typically colloidal polymer films confining metabolically active, nongrowing bacteria. Depending on the species of confined bacteria, biocoatings find applications in wastewater treatment, biofuel production, carbon fixation, environmental remediation, biosensing, and more. However, the successful use of biocoatings faces numerous challenges, including a low permeability to reactants and metabolized products, osmotic stress on bacteria during drying of the coatings, and cell dehydration leading to bacteria death. Here, we address these challenges through two interlinked processing methods. (1) Coagulant gelation of the colloidal polymer dispersion creates a porous microstructure with high permeability. (2) Wet sintering by immersion in a liquid medium reduces osmotic stress and avoids desiccation of the bacteria. In a model system of *Escherichia coli* in an acrylic copolymer latex biocoating, these two methods yielded a cell viability that is approximately 500 times greater than the conventional method of biocoating formation using dry sintering in air at an elevated temperature. We have discovered that when lysogeny broth is used as the medium for wet sintering, the cell viability is significantly higher than that for other liquids. Increasing the salt concentration for coagulant gelation leads to thicker coatings (and hence more cells per area of the coating). However, cell viability decreases when the salt concentration is increased, so a compromise is needed. Metabolic activity of *E. coli* in a wet-sintered biocoating was demonstrated through the production of ethanol as a biofuel. These results hold promise for the future exploitation of biocoatings using a broad range of bacterial, especially desiccation-intolerant species.

## Graphical abstract

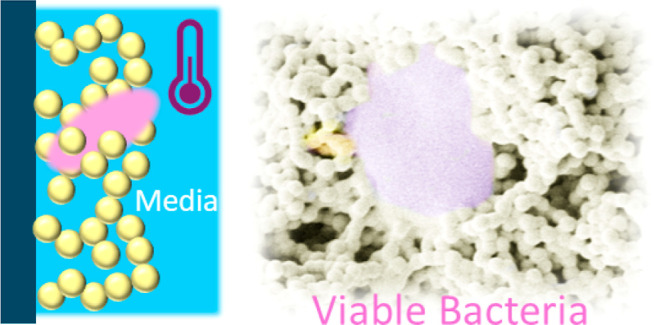

## Introduction

Natural biofilms are communities of living bacteria that are embedded in a self-produced polymeric matrix and are attached to a surface or to each other.^1,2^ Synthetic biofilms, called biocoatings, are usually made by mixing live bacteria with a stable dispersion of colloidal polymers in water^3^ and then drying to make a solid coating.^4^ The bacterial cells are encased within the coating, alive but nongrowing. The metabolically active bacteria in biocoatings have found applications in wastewater treatment,^5,6^ carbon capture^7,8^ indoor air purification,^9^ biofuel synthesis,^10,11^ biocatalysis,^12^ and environmental remediation.^13^ In the last year, *Nature* published a “call to action”, “demanding immediate, tangible steps that harness the power of microbiology”.^14^ The paper highlighted the “great promise” of microbe-based technologies to combat the escalating issue of climate change. Biocoatings provide an excellent means to contain, to protect, and to transport bacteria for these purposes.

The genome of *Escherichia coli* (*E. coli*) was fully sequenced nearly three decades ago, and *E. coli* has been a frequent subject of synthetic biology, leading to its application in antibiotic production,^15^ synthesis of biofuels^16–21^ and aromatics,^22,23^ and biosensors.^24^ Lab-adapted *E. coli* is an ideal model organism and is therefore used in this research.

Conventionally, the film formation of biocoatings is achieved from mixtures of aqueous suspensions of bacteria with polymer colloids in water (known as latex dispersions). After film casting, water evaporation increases the concentration of the colloids, leading to packing of the particles. The particles undergo deformation to reduce interfacial energy (in the process of sintering), which eliminates void space between them. Finally, the interdiffusion of polymer molecules between particles (coalescence) results in a cohesive and mechanically robust coating. Despite the diverse functionality and utility of biocoatings, there are numerous challenges that have prevented their more widespread use. The polymeric binder of a biocoating must ensure viability during film formation and then physically confine the bacteria, provide mechanical protection, and allow chemical exchanges with the surrounding environment. Presently, the most urgent materials-related problems of biocoatings are (1) low porosity and permeability,^25^ (2) high osmotic stress on bacteria during film formation,^26^ and (3) bacterial cell dehydration leading to reduced viability.^27^

### Need for High Permeability

Biocoatings must have sufficient permeability to allow the ingress of nutrients and reactants to allow cells to function, and also to allow the egress of reaction products and metabolites out of the coating.^26^ Flickinger et al. found that stable film porosity is necessary to optimize the activity of the bacteria in a biocoating.^26^ A common strategy to retain void space in biocoatings is the inclusion of hard fillers, such as glassy polymer particles^28^ or halloysite clay,^4^ to arrest the particle coalescence and to introduce nanoscale gaps within the coating. A related approach is to fill the interparticle voids with nonvolatile, water-soluble molecules, such as glycerol,^29^ sucrose^25^ and carbohydrates,^30^ as porogens. These phases will block colloid particle contacts, and then can be rinsed out after film formation to leave behind an air void. As an example, the addition of sucrose to a latex formulation was found to increase nitrate diffusivity by a factor of 35.^28^ However, the voids are not permanent when the coatings are stored at temperatures above the glass transition temperature, *T*_g_, of the polymer binder. Experiments have found that gradual particle deformation and coalescence still occur slowly over time, so that the volume of the void network (and permeability) decreases.^25,31^

### Need to Reduce Osmotic Stress

Flickinger et al. identified that minimizing osmotic stress is crucial to preserving bacterial viability.^26^ Osmotic stress occurs when the concentration of species in the aqueous solution surrounding bacteria differs greatly from what is inside the cells.^32^ When bacteria are in high concentrations of aqueous salt solution, water will diffuse out of the cells, causing them to experience dehydration. Alternatively, when cells are placed in deionized water, there will be a net transfer of water into them, causing their membranes to rupture.

In the past few years, methods have been developed to reduce the osmotic stress during the film formation process. For example, Gosse et al. developed a method in which the colloidal dispersion is absorbed into chromatography paper and kept hydrated.^33^ The drying step was avoided entirely, but cells adhered to the substrate. Gosse et al. found that their coatings retained cell reactivity as measured by oxygen gas evolution.

### Preventing Cell Dehydration

When biocoatings containing bacterial cells are placed in air, the removal of cell-bound water results in structural, physiological and biochemical stresses.^27^ In desiccated coatings, the residual water is often not sufficient to maintain even a monolayer of water around the extracellular macromolecules. Metabolic enzyme-catalyzed reactions, which require water, are not possible in this environment. Therefore, dried bacterial cells do not remain viable.

Only a subset of bacteria are anhydrobiotic, with the ability to withstand removal of water.^27^ Desiccation tolerance is the ability of this subset of bacterial species to undergo severe dehydration through air drying without being killed. Traditional methods for the creation of biocoatings include a drying step as part of the film formation process. In order to maintain the viability of bacteria immobilized within the coating using these methods of film formation, desiccation tolerance is required. Note that desiccation stress on bacteria is a general term that can refer to osmotic stress and also to cell dehydration, both arising when bacterial cells are exposed in air, and water is removed.

Previous research on engineering desiccation tolerance in bacterial species has studied the mechanisms that anhydrobiotic bacteria use to withstand desiccation. For example, Billi et al. used sucrose-6-phosphate synthase to increase the survival rate of*E. coli* under air drying, desiccation and freeze-drying.^34^ Sucrose and trehalose are nonreducing disaccharides which act to protect bacterial membranes and proteins during desiccation. By maintaining a supply of trehalose or sucrose through the desiccation process, bacterial cells were found to have a greater probability of survival.^35^ To maintain this supply, sucrose or trehalose may be added extracellularly^35,36^ or synthesized internally.^34^ However, Chen et al. found that the addition of trehalose or other carbohydrates to biocoatings did not significantly increase the viability of confined*E. coli*.^4^

Swope and Flickinger correlated the extent of cell drying with a loss of viability.^29^ Cells in biocoatings that had been dried for 1 h were 70–80% viable, whereas an added rehydrating step yielded a viability of 95%. They suggested that the cellular membranes of immobilized cells weakened during drying.

In a second study during the same year, Swope and Flickinger investigated the regeneration of *E. coli* bacterial cells within biocoatings after a period of starvation, finding that the immobilized cells could be reinduced for up to 17 days after the initial induction, with viability remaining >90%. (Here, reinduced refers to the addition of isopropylthiogalactoside, an inducing agent, to bacteria that were previously in a starvation medium.)^37^ With this work, they demonstrated that periodic induction is a valuable method for extending the life of biocoatings.

Recently, Chen et al.^6^ reported a film formation process in which evaporation was suppressed to keep cells hydrated after the colloidal particles were deposited in a packed layer. Chen et al.^6^ coagulated charge-stabilized colloids by spreading the dispersions on salt-coated substrates, in a method inspired by the latex glove-making process.^38^ Colloidal particle coalescence took place in the presence of an unquantified amount of aqueous solution. We will refer to the mechanism hereafter as “moist sintering” to recognize the presence of both condensed water and air during the process.^39^ The reactivity of the nitrifying bacteria in their moist-sintered biocoatings was higher. The biocoatings produced five times more nitrites and nitrates than was found for conventionally dried biocoatings.

Both Gosse et al.^33^ and Chen et al.^6^ found that film formation under conditions of high relative humidity was beneficial to the viability of the encapsulated bacteria. This technique of “moist” sintering is a promising method to avoid dehydration and so increase viability.

### Proposal for Coagulant Dipping and Wet Sintering by Immersion

To address the problems faced by biocoatings, we have drawn upon two key concepts of colloid science: coagulant gelation and wet sintering.

Coagulant gelation is employed routinely in the manufacture of latex gloves and balloons. An appropriate mold is coated with a salt, such as Ca­(NO_3_)_2_, before being submerged into a charge-stabilized latex dispersion. At positions near the interface with the mold where the local ionic concentration exceeds the critical coagulation concentration (C*), a gel is formed. Groves and Routh developed a model to describe the growth of the gel layer, which considered the diffusion distance of salts over time to define the range of distance where C* is exceeded when dipping into latex.^38^ Williams et al.^40^ provided an analytical solution to the diffusion model and derived an equation for the thickness of the gel coating as a function of the amount of salt on the substrate, the diffusion coefficient of the salt in water, and the C* of the salt. Their model predicted that at longer dipping times, the thickness of the gel coating is directly proportional to the amount of salt on the substrate. Experimental data validated the model.^40^

Under wet sintering, colloids in a packed array will deform to fill space as a means to reduce their interfacial area with the water, thus reducing their free energy. The mechanism was first proposed by Vanderhoff.^41^ Experimentally, Sheetz concentrated polymer colloids in dialysis bags to make agglomerates in water. He used gravimetric methods to examine the increase in solids content as the particles underwent wet sintering.^42^ After a film is deposited on a substrate, wet sintering can be achieved by suppressing water evaporation when the particles are in close contact. The time scale for particle deformation in water is then shorter than the time for water loss, as is required by Routh and Russel’s deformation model.^43^ In experiments, Dobler et al.^44^ found that coalescence of particles in water followed a first-order kinetic relationship with respect to the water content. A faster rate of coalescence was obtained for higher water temperatures, when the polymer viscosity was lower.

Physical contact points between colloidal particles in the presence of liquid water can be achieved following the concentration of particles locally via water evaporation, dialysis or coagulation to enable wet sintering. In this research, we have created biocoatings using the process of coagulant gelation by dipping into a suspension of bacteria in latex. The gel coatings are then wet-sintered by immersion into a liquid medium. The main stages of our proposed process are presented in Figure 1. Our technique is based on the coagulant dipping process used in the latex glove-making industry,^38^ but a key difference is that we use colloids in their glassy state at the temperature of dipping, rather than the elastomeric particles used for rubbery products. Figure S1 (Supporting Information) shows photographs of the experimental setup.

**Figure 1:**
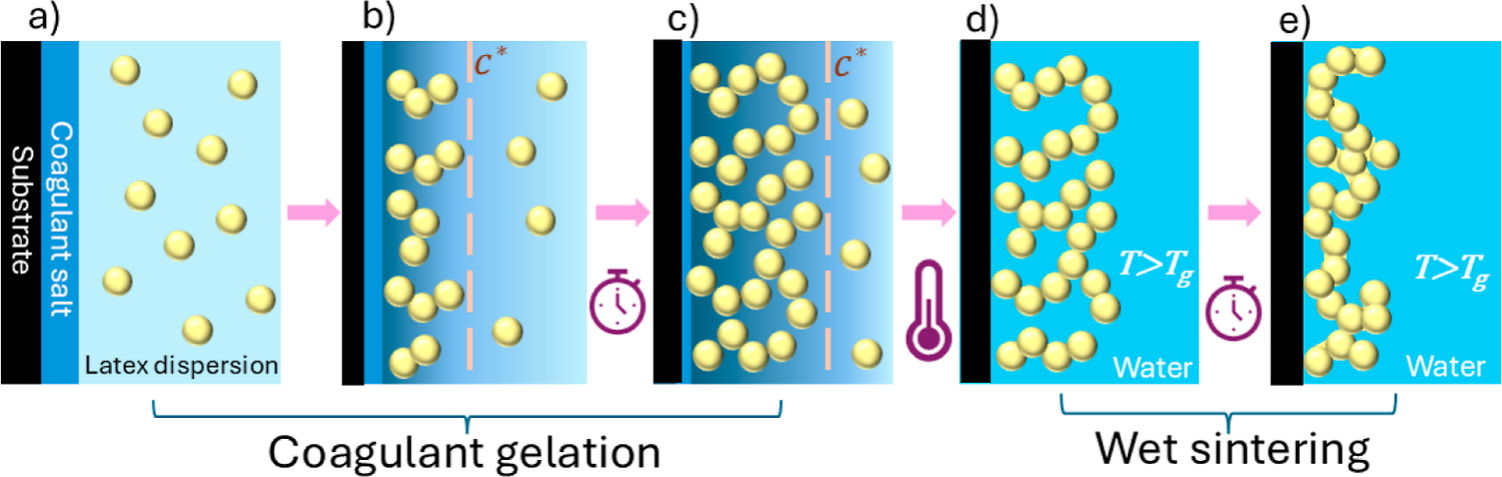
Stages of the methods of coagulant gelation and wet sintering. (a) A substrate coated with a coagulant salt is in contact with the initial latex dispersion. (b) The salt diffuses into the aqueous phase, and the latex coagulates at distances where the concentration exceeds C*. (c) The thickness of the coagulated gel layer increases over time. (d) The gel coating is immersed in water at a temperature above the polymer’s *T*_g_. (e) The particles undergo wet sintering to create a mechanically robust structure.

The key innovation in our process is following the gelation with wet sintering by immersion in a warm liquid. After the coagulant-coated substrate is dipped into the latex dispersion and withdrawn, the resulting gel coating is immediately immersed into a liquid at an elevated temperature (above the polymer’s *T*_g_) for a set period of sintering time. At the completion of film formation, the coatings can be stored in the medium at a temperature below the polymer’s *T*_g_, where they remain mechanically stable.

This processing strategy has numerous benefits. First, it allows the use of a latex with a higher *T*_g_ (above the ambient temperature) while avoiding the drying step entirely. Hence, the problem of capillary stress and cracking,^45^ when a coating is dried in an oven, is prevented. Crucially, the negative effects of osmotic and desiccation stresses are minimized because the drying stage is completely avoided, and the sintering liquid can be selected to suit the bacteria. Hence, the strategy holds potential to increase the viability and longevity of bacteria.

## Results and Discussion

### Establishing the Conditions for Wet Sintering by Immersion

We used a charge-stabilized acrylic copolymer latex (*T*_g_ of 28 °C), synthesized with a low surfactant concentration to reduce toxicity to bacteria. The latex has a negative zeta potential and a critical coagulation concentration of ca. 0.07 M, which made it suitable for coagulant dipping. Calcium nitrate tetrahydrate was chosen as the coagulant salt because it is sometimes found in growth media and can be well tolerated by bacteria. This coagulant was deposited on paper substrates at an areal density (ρ_c_) of 0.055 mol/m^2^. When the salted substrate was dipped in latex with a low solids content (14 wt %), a gel coating with a thickness greater than 200 μm was deposited. When the concentration of salt on the substrate was increased, the thickness of the gel coatings likewise increased (Figure 2a). This correlation is consistent with models of coagulant dipping presented previously.^38,40^

**Figure 2:**
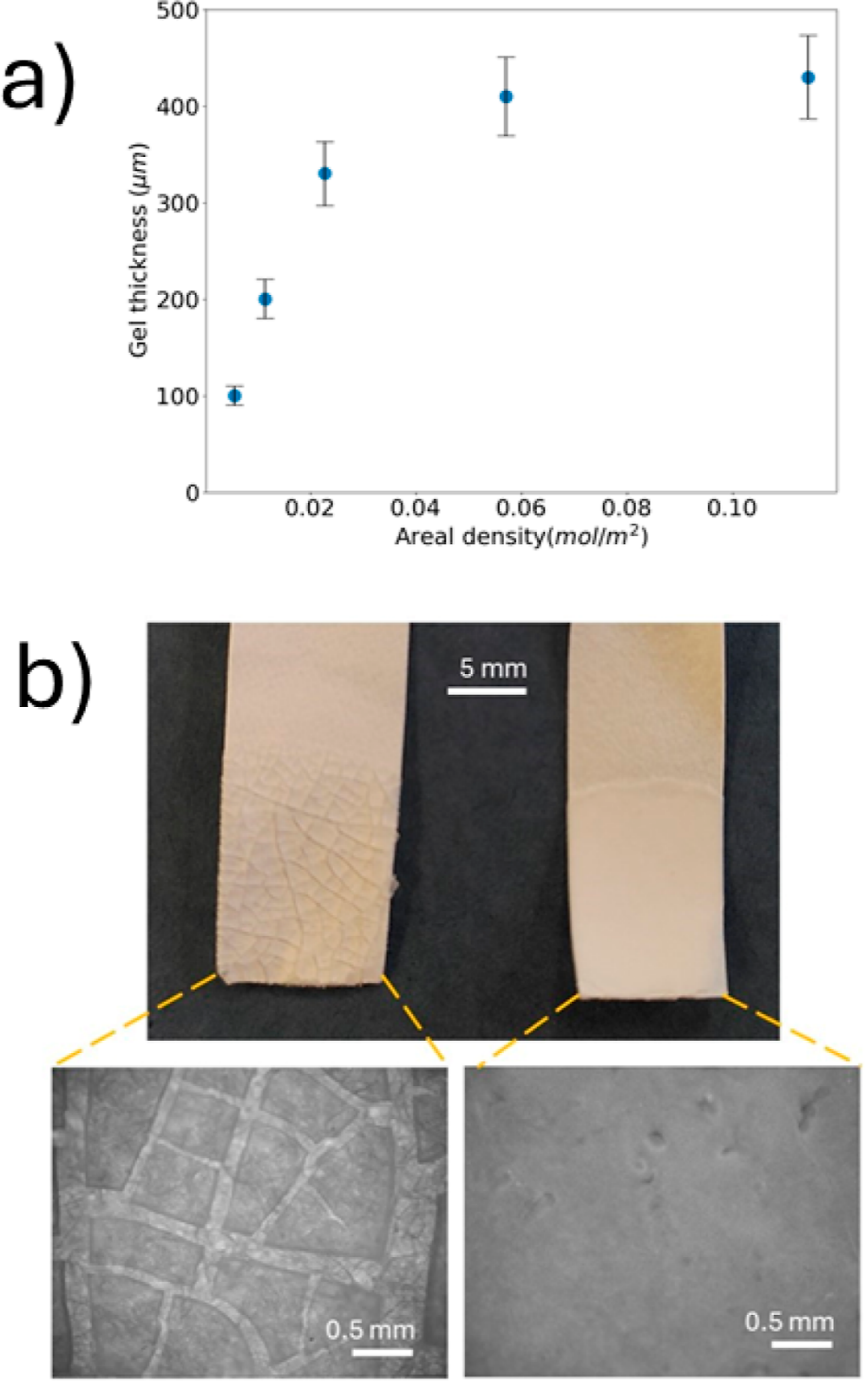
(a) The dependence of the thickness of colloidal gel coatings on the areal density of (Ca­(NO_3_)_2_) on the substrate used for coagulant dipping. The error bars represent the uncertainty of the measurements. (b) Visual comparisons of the methods of sintering of a coagulant-dipped coating at an elevated temperature of 35 °C via (left side) dry-sintering in a convection oven and (right side) wet sintering in water for 3 h. The images in the top row are photographs of the coatings; in the bottom row, the images are obtained via optical microscopy. In both images, the coating covers the lower half of the substrate; the upper half is bare paper.

We found that the coagulation step was essential to achieve a sufficiently thick coating via wet sintering by immersion. In the absence of a coagulating salt on the substrate, a robust coating could not be deposited. Upon immersion of a dried, nongelled colloidal layer in water, the colloidal particles readily redispersed in the water. Very few colloidal particles adhered to the paper substrate (see the SEM images in Figure S2 in the Supporting Information).

When the colloids are dried in the absence of coagulants, attractive van der Waals forces are operative, but there is still an opposing charge repulsion between the negatively charged colloids. However, when the colloids are coagulated, the divalent Ca^2+^ ions can bridge the negatively charged colloids in close contact, which will build cohesion in the gel structure.

An example of a coating formed by coagulant dipping followed by wet sintering by immersion in water is presented in Figure 2b. For comparison, a coating made via “dry sintering” the gel layer in air in a warm oven (35 °C) is also shown. This comparison highlights the benefit of avoiding the drying step during film formation. The dry-sintered coating displays severe cracking, which is attributed to the effects of capillary stresses when the colloidal particles are in a packed bed.^45–47^ As the heat transfer from air in an oven is inefficient, the particles were likely to be in the glassy state during the initial stage of water evaporation. In contrast, the wet-sintered coating is continuous and defect-free. Water was not removed until after the particles had coalesced in warm water, and after polymer interdiffusion between the particles had strengthened the colloidal network.^47^ The benefit of wet sintering to film quality is apparent here.

Upon successful film formation, the coatings are in a hard, glassy state at room temperature, as confirmed by pendulum hardness testing (Table S1 in Supporting Information).

Furthermore, we found that the wet sintering process needed to take place in a liquid with a temperature above the acrylic copolymer’s *T*_g_ of 28 °C. When coagulant-gelled coatings were immersed in water at a temperature of 23 °C, the resulting coatings were brittle and severely cracked. See Figure S3. This result illustrates how particle coalescence is only possible at temperatures above the polymer’s *T*_g_ when it can flow during the film formation process.

Scanning electron microscopy of the coatings revealed a porous structure created from a loose packing of the colloidal particles, with the particles fused at their contact points (Figure 3a). This microstructure was stable when the coatings were stored at a temperature below the copolymer’s *T*_g_ for 1 week (Figure 3b). A frequently cited problem with softer coatings is their lack of microstructural stability,^39,43,48–50^ but our method has been found to overcome this issue. The porous microstructure can be seen throughout the cross-section of a coating with a thickness of 30 μm (Figure 3c,d). When a coating was debonded from a paper substrate, it was observed to be conformal to the rough fiber surface (see Figure S4). Upon debonding, some fibers were removed by the coating, indicating strong adhesion, which is a requirement for biocoatings in applications. Moreover, we found that varying the substrate type does not have a noticeable effect on the resulting microstructure. See Figure S5 in the Supporting Information.

**Figure 3:**
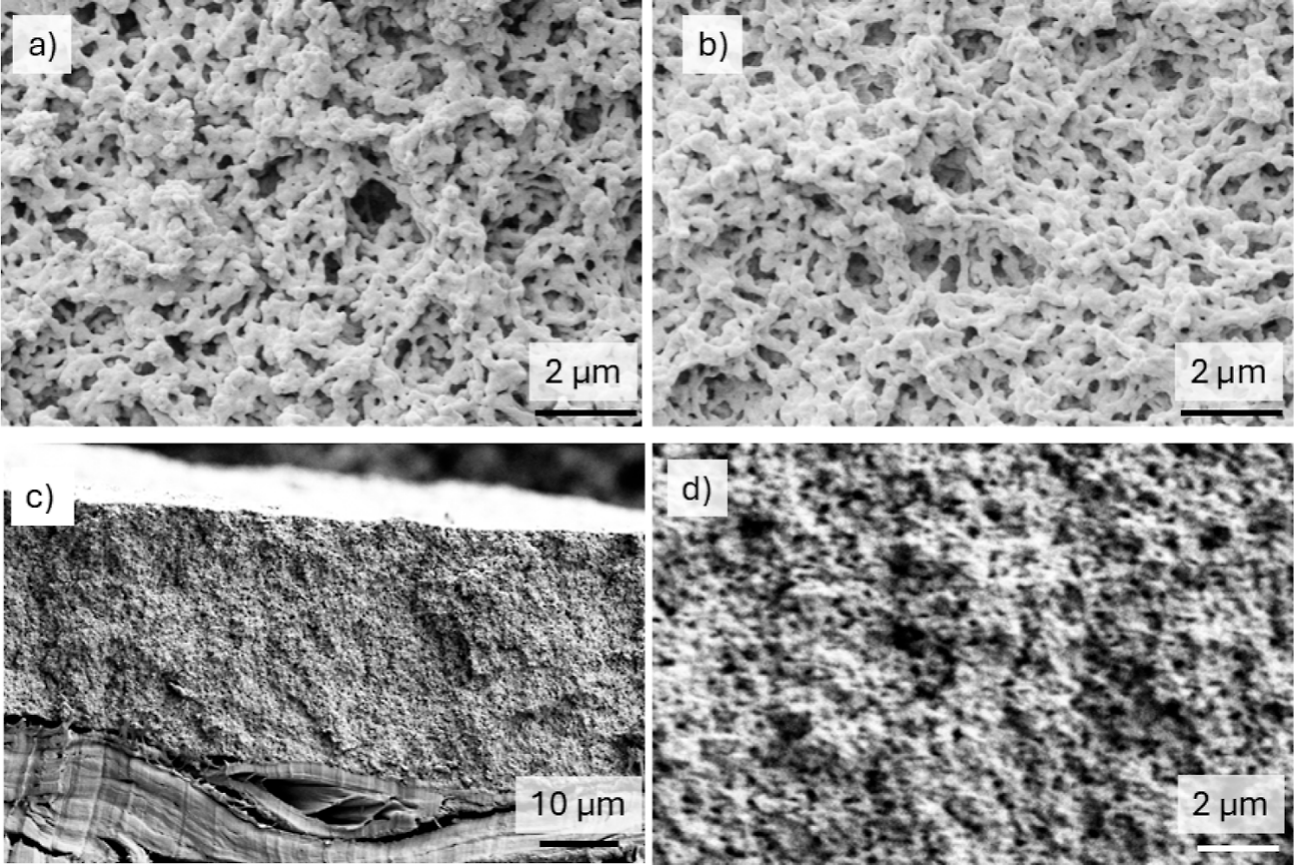
SEM images showing the microstructure of coatings. (a) Top surface of a coagulant-dipped coating made by wet sintering in water using an immersion time of 3 h and a temperature of 35 °C, shown on the day of film formation. (b) The same coating imaged after storage at approximately 40% relative humidity (RH) and a temperature of 21 °C for 7 days. (c) The cross-section of a coagulant dipped coating after wet sintering by immersion in water for 30 min at 34 °C. The paper substrate can be seen at the bottom. The film surface is along the top of the image. (d) Higher magnification of (c) providing evidence for voids (appearing as dark spots) throughout the depth of the coating.

The porous microstructure yielded a high permeability to water. We investigated the impact of the microstructure on the liquid water permeability using a gravimetric method. See the data in Figure S6. The water permeability of a coagulant-dipped, wet sintered coating (in water at a temperature of 32 °C for 30 min) was found to be 7.2 ± 2.3 μmol/m/s. For comparison, the value obtained from a dry sintered acrylic coating was more than an order of magnitude lower with a value of 0.55 ± 0.09 μmol/m/s. The higher permeability of the coagulated, wet-sintered coating correlates with the porous structure observed with electron microscopy.

### Parameters Affecting Microstructure and Density of Coatings

We explored the parameters that can be used to tailor the wet-sintered coating microstructures to make them suitable for applications in biocoatings. First, we present the effects of the temperature of the water used for immersion.

Figure 4 shows electron micrographs obtained from varying the temperature of the water in which the sample was immersed. The images were obtained for an immersion time of 30 min for four different water temperatures. At the lowest water temperature (29 °C), the individual colloidal particles can be observed with a thin neck at their contact points. At a wet sintering temperature of 35 °C, the particles are more deformed, and are no longer distinguishable, forming chain-like structures. At higher temperatures of 49 and 59 °C, there is a noticeable change in the microstructure. Larger voids appear between clusters of coalesced particles, see Figure 4c,d. There are particularly large voids (on the order of 2 μm) at the highest temperature. Measurements of the coating thickness for increasing sintering temperatures reveal that the structure of the colloidal gel collapses more as the sintering temperature is increased (Figure 5). However, thicknesses of coatings sintered at the two highest temperatures are the same, which suggests that there is void growth by the fusion of smaller voids, without a simultaneous collapse in the structure.

**Figure 4:**
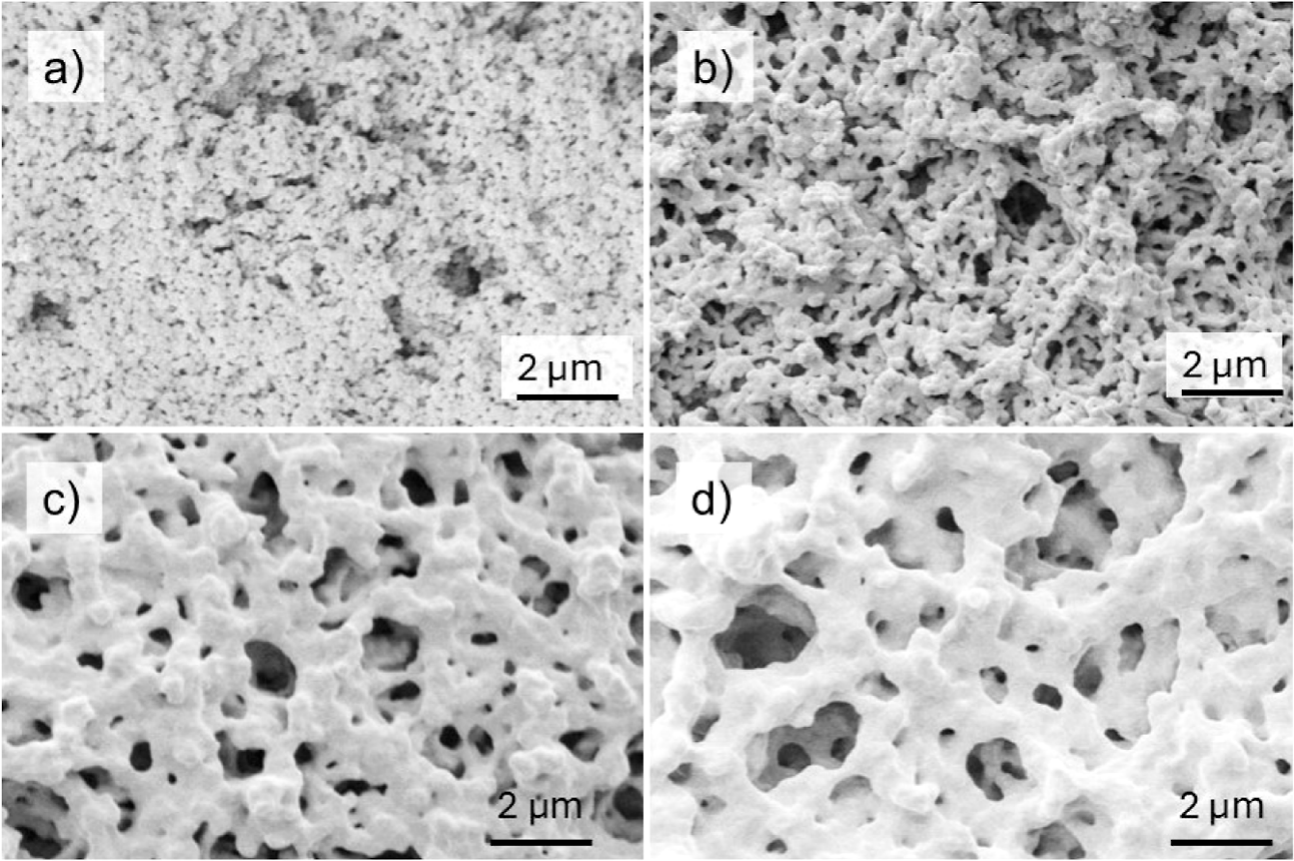
SEM images showing the effect of the temperature of the water for immersion on the resulting microstructure of the coating: (a) 29 °C, (b) 35 °C, (c) 49 °C, (d) 59 °C. The samples were immersed for 3 h.

**Figure 5:**
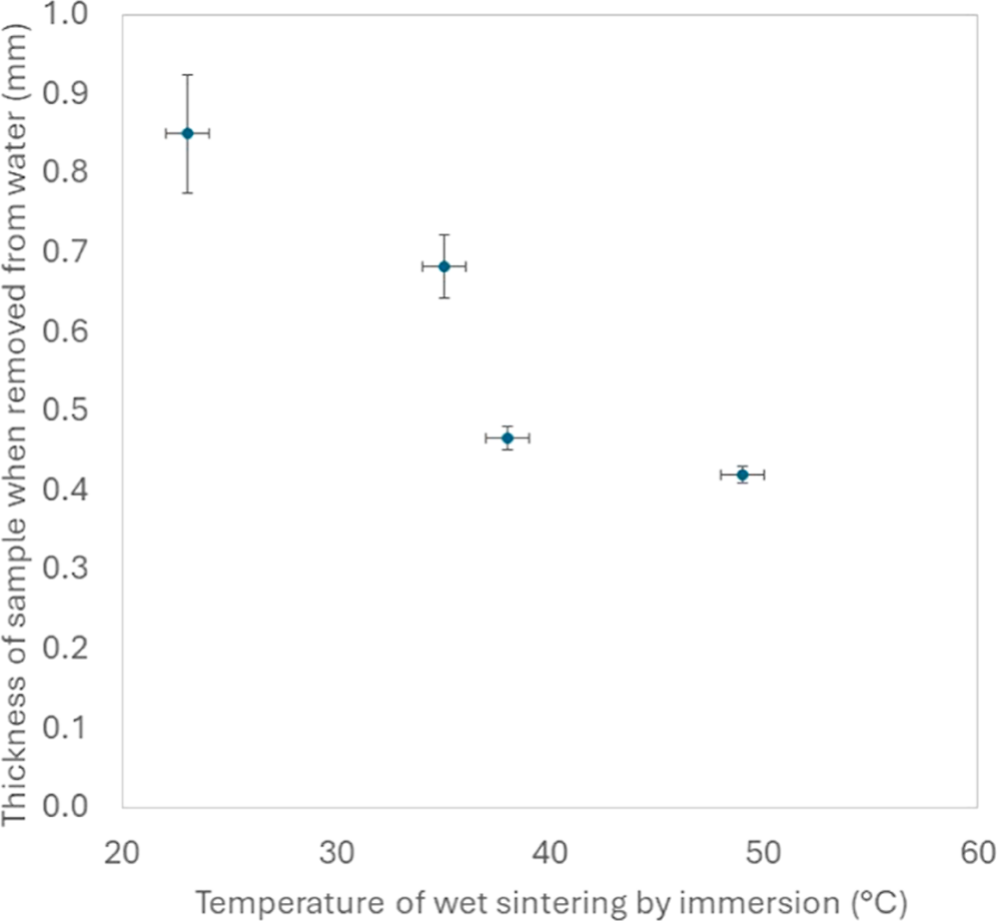
Wet thickness of the wet-sintering coatings after an immersion time of 3 h as a function of the immersion water temperature. The error bars on the thickness show the standard error in the mean from three replicate thickness measurements per sample.

Next, we explored how the colloidal coating microstructure evolves over time. Typically, the sintering of particles in a packed bed leads to a gradual decrease of the void size and an accompanying increase in the density.^51,52^ However, in our colloidal gel structures, in which the particles are not initially densely packed, coarsening of voids is observed. The time of immersion in deionized water was found to have a significant impact on the microstructure of the coatings, as can be seen in the SEM images in Figure 6. At the shortest immersion time of 2 min, spherical particles form a neck region with their nearest neighbors to create short strings. Further widening in the neck regions is observed at longer immersion times so that the particles lose their identities. At the longest immersion times, there are fewer voids apparent between clusters of particles. Instead, voids appear to grow in the network of sintered particles, rather than shrinking in size.

**Figure 6:**
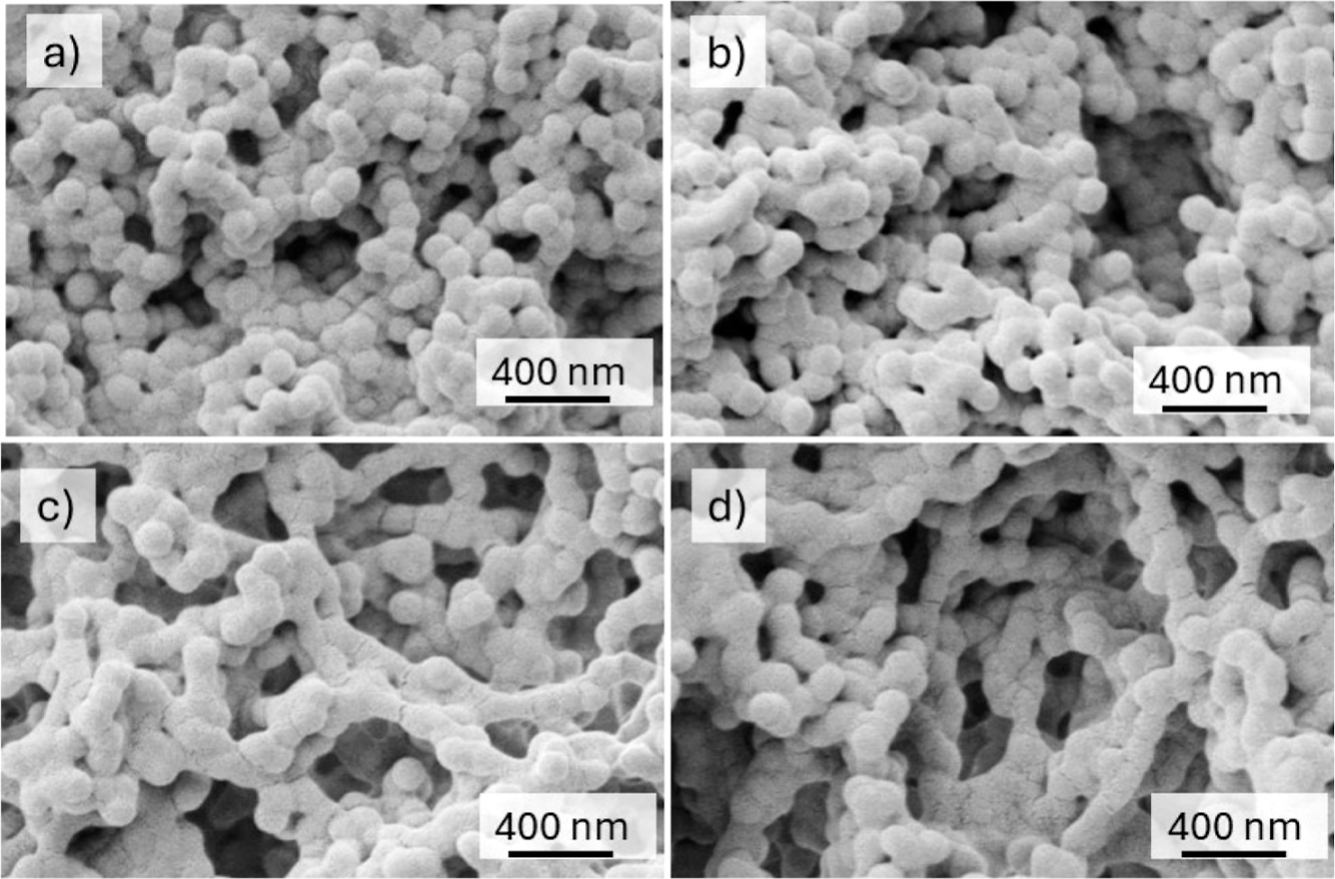
SEM micrographs showing the effect of the wet sintering immersion time on the microstructures of the colloidal coatings: (a) 2 min; (b) 15 min; (c) 30 min; and (d) 45 min. The temperature was 35 °C in all experiments.

Analysis of a large number of SEM images for a range of experiments has shown that the typical void size increases with the time of sintering by immersion in water. Image analysis was performed on the micrographs in Figures 4 and 6 (along with 13 other samples not shown here) to extract a mean diameter of voids visible at the surface of the coatings (see Figure S7 in the Supporting Information for details of the image analysis).

The driving force for sintering is the reduction in the surface free energy through a decrease in the interfacial area between particles and the surrounding medium (air or liquid). The free energy is found from the product of the area and the interfacial energy, γ. For viscous particles, the flow to reduce the area is resisted by the viscosity, η. Considering these parameters in sintering models^43,53^ for particles with a radius of *a*, the dimensionless time for sintering, τ, is given as γ*t*/η*a*. For polymers, the viscosity is dependent on the temperature above the *T*_g_. We employ the well-known WLF equation^54^ to obtain an estimate of the polymer viscosity at each of the sintering temperatures. We then consider the effect of the temperature on void growth by using that viscosity to find the dimensionless sintering time. Figure 7 presents the dependence the void radius on τ in experiments where either the wet sintering time or the sintering temperature was varied. The dimensionless value of the mean void radius, r*, was obtained by dividing the void radius, *r* by the mean particle radius, *a*. The temperature dependence of γ was neglected when calculating τ, and a constant value of 10 mJ m^–2^ was always used. The data are presented in Figure 7.

**Figure 7:**
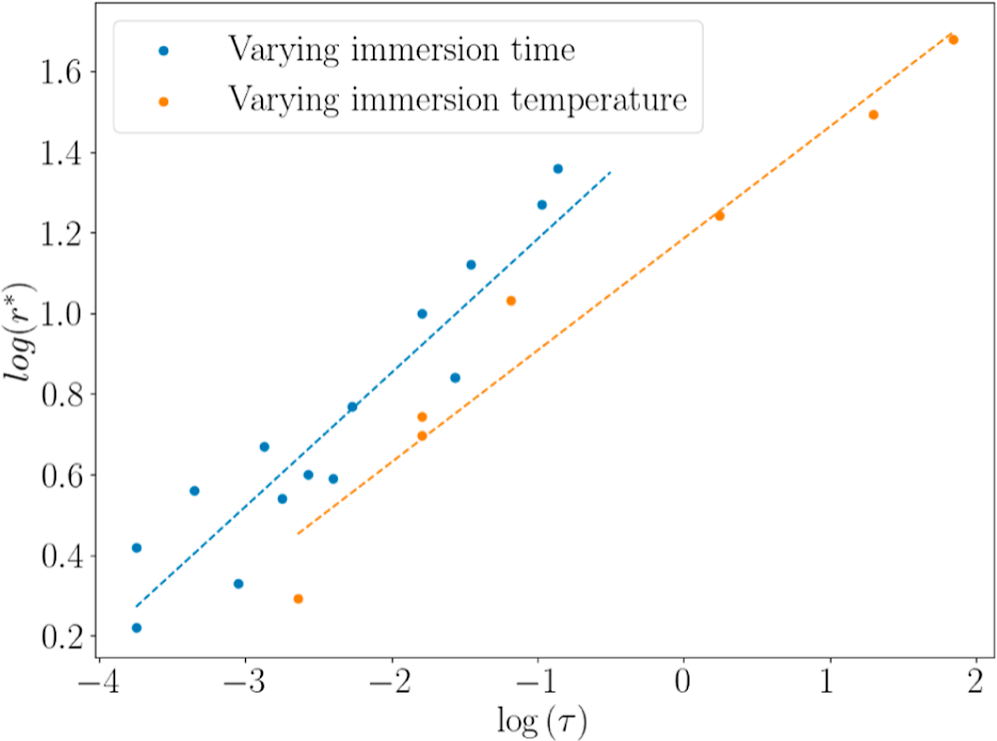
Dependence of the dimensionless radius of the voids, r*, in the microstructures of latex gel coatings on the dimensionless time, τ. The data points show an average radius of the four largest voids, using two images per sample.

We propose that the voids grow over time by the fusion of smaller voids between individual particles. A larger void has a higher surface area than a small void. But because a large number of smaller voids will disappear when they fuse into a single larger one, the overall surface area of the microstructure will be reduced. We draw an analogy to coarsening during the spinodal phase separation of viscous fluids.^55–57^ In Figure 7, varying either the immersion time or the temperature reveals a similar positive correlation with the void size. These data show that the void radius scales approximately as ∼τ^0.3^. (The gradient of the line of best fit for varying immersion times is 0.33 ± 0.16, whereas it is 0.27 ± 0.09 when varying the temperature.) We draw the conclusion that the microstructure of the coatings can be tailored in a precise way through the immersion conditions during wet sintering.

Nawaz and Rharbi^48^ studied sintering in latex films using small angle neutron scattering (SANS) and likewise found evidence for coarsening. For particles smaller than 60 nm diameter, for which there was no close packing, they observed the growth of voids. Their measured peak wavevectors in SANS provide an estimate of the void sizes. After extracting values presented by Nawaz and Rharbi for a range of film formation times, we investigated the time dependence of coarsening. Specifically, from our own analysis of their data, we found that their voids increased in size as ∼*t* ^0.4^ for 30 nm particles and as ∼*t* ^0.2^ for 42 nm particles. Our τ^0.3^ scaling is intermediate to these two relationships. Considering the experimental uncertainties, the same mechanism could be at play here.

We propose that the void growth observed in the gel coatings arises because there is an initial loose, random packing of the particles obtained by coagulant gelation, rather than a hexagonal close-packed arrangement of particles. Indeed, Nawaz and Rharbi commented that those particles that exhibited coarsening were randomly packed because of their dispersity in size.

### Investigation of the Densification of the Coatings

To gain further insight into the film formation of the coagulant-dipped coatings, their relative densities were estimated over time. The density of the coating was estimated from the measured mass of the coating, its area, and its thickness obtained from photographic images of the cross-section. The relative density is defined as the estimated coating density divided by the density of the copolymer (taken to be 1130 kg/m^3^) (see the Supporting Information Text and Figures S8 and S9 for a description of the method).

When a coagulant-dipped coating is placed in water at room temperature, the gel structure does not collapse. Instead, the initial relative density of 0.1 remains relatively constant, as is shown by the blue line in Figure 8. This result suggests that the hard particles, which are not able to undergo wet sintering, do not displace the surrounding water by collapsing the gel structure. Instead, the low density of particle packing is retained. When the temperature of the water is raised to 32 °C, which is above the polymer’s *T*_g_, a greater amount of densification is observed. There is an initial rapid densification followed by a leveling out of the densification rate.

**Figure 8:**
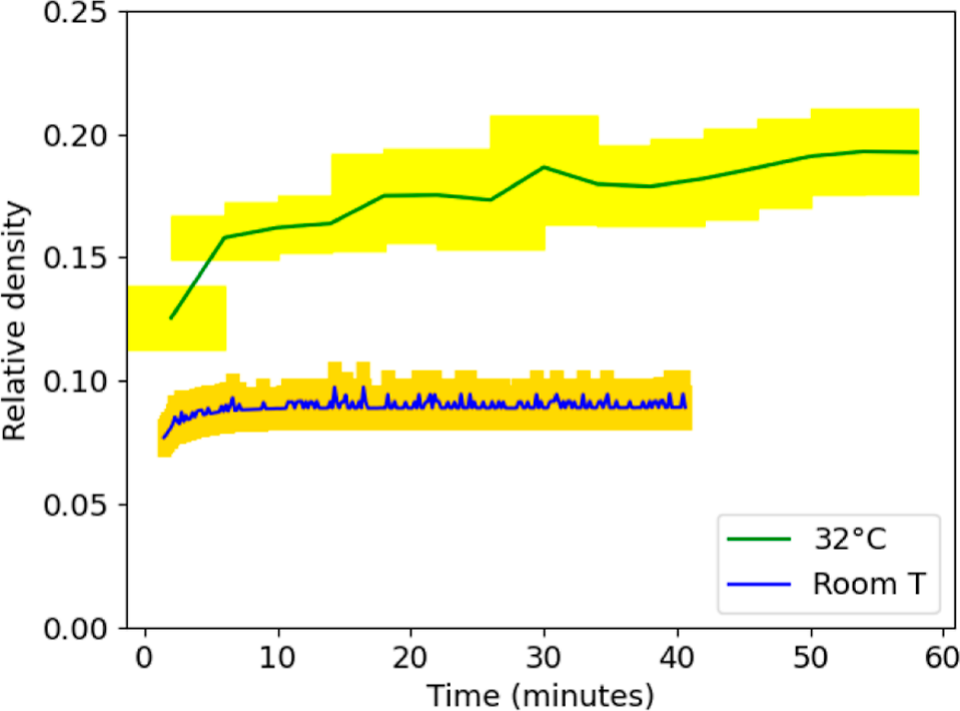
Relative density of gel coatings immersed in water at the room temperature (24 °C), which is below the polymer’s *T*_g_ (blue line) and at an elevated temperature (∼32 °C, green line), above the *T*_g_, as a function of time. The yellow region displays the uncertainty in the measurements from two or three replicate experiments.

The microstructures observed using SEM provide insight into the interpretation of the densification data. At short times (up to 15 min), particle deformation and neck growth were observed. The effect of this sintering is to reduce the thickness of the coating, and thereby increase the density, according to established models of sintering.^43,51,53^ During this time period, the relative density increases from approximately 0.12 to 0.18. Beyond 15 min of immersion in water where the density increases only gradually (an increase of about 0.02 in 45 min), the SEM analysis showed the growth of voids. If there is fusion of small voids between packed particles or between strings of particles, the density of the structure is not expected to change. The observed slow rate of densification can be explained by the volume of individual voids increasing while the number of voids decreases as a result of their fusion. (In contrast, we found that if the coagulant gel coatings are stored in air at room temperature, the hard particles gradually collapse into a dense packing during drying. See Figure S10.)

We conclude that the early stage of film formation in water is dominated by the wet sintering of adjacent particles. The later stage is dominated by the coarsening of the voids. Figure 7 is primarily showing the coarsening region of the process.

### Cell Viability in Biocoatings

#### Microstructure of Biocoatings

Nonpathogenic, laboratory-disabled *E. coli* bacterial cells were successfully confined within biocoatings via coagulant gelation and film formation conducted aerobically. Figure 9 shows an SEM image of bacterial cells in latex coatings. Two types of sintering are compared here: dry sintering and wet sintering by immersion. To achieve dry sintering, coagulant-dipped gel coatings were placed in a convection oven at a temperature of 35 °C. When the water evaporated, the interfacial energy of the polymer/air interface was the driver for sintering. In the dry-sintered biocoating, latex particles in a close-packed arrangement surround the bacterial cells within a closely packed structure (Figure 9a). The largest open spaces arise from the microcracking of the material. Cracks may be observed as the fracture lines stretching from the top to the bottom of the image. In the regions without a crack, the average void size is approximately one-third of the projected area of a latex particle. A network of small voids provides some porosity. Figure 9b provides a high-magnification micrograph of an example individual bacterium in the dry-sintered coating. The latex particles surround the perimeter of the bacterium in a closely packed line along the cell periphery, and there are some localized regions of ordered particles surrounding it.

**Figure 9:**
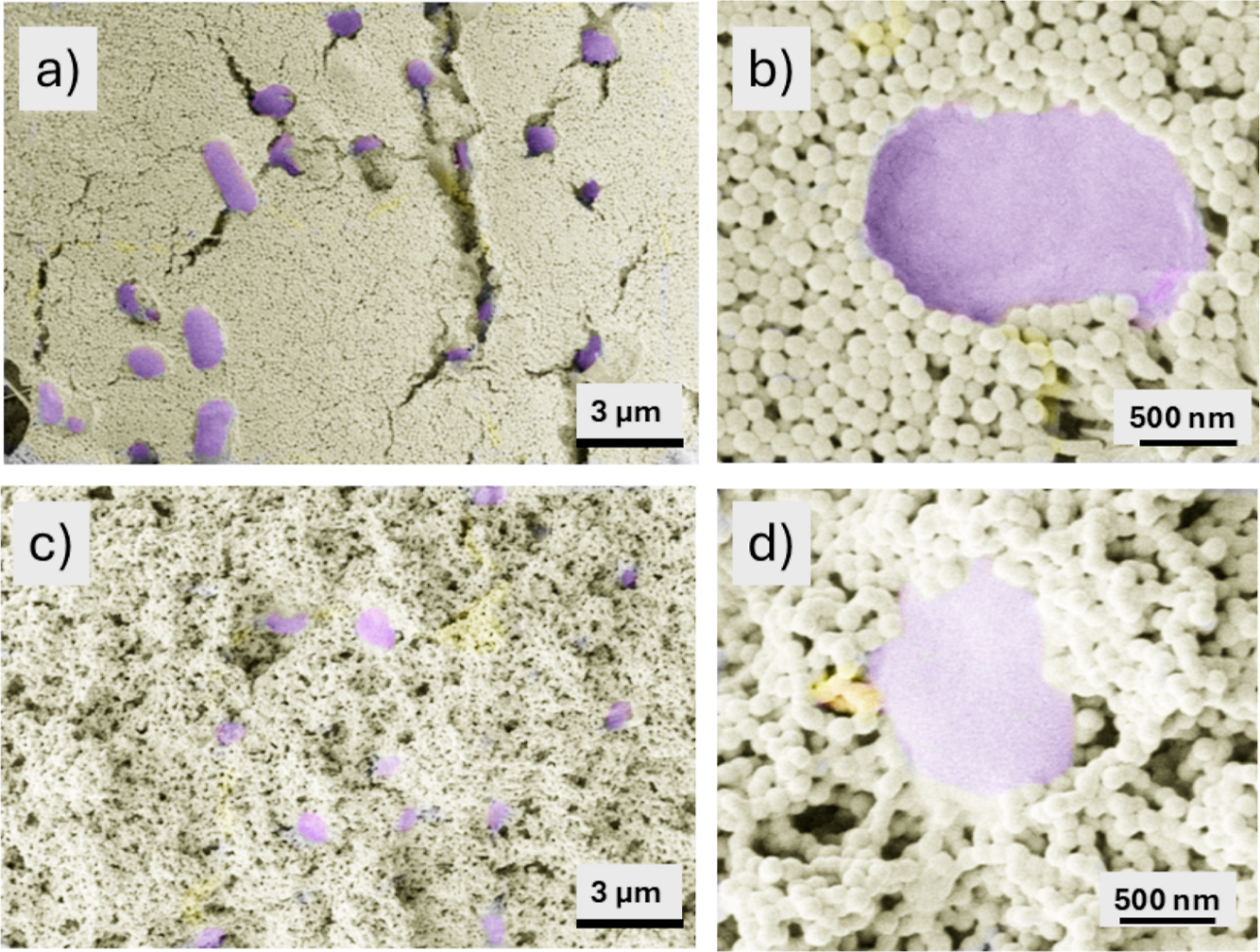
SEM micrographs of the top views of biocoatings showing *E. coli* bacterial cells (false-colored purple) encased within a colloidal coating (latex particles shown in yellow). (a,b) A dry-sintered biocoating, created using a gel film-formed at 40% RH in an oven; (c,d) a biocoating formed using the technique of wet sintering by immersion in PBS.

In a biocoating created via wet sintering by immersion in phosphate buffered saline (PBS) (Figure 9c), more porosity is observed at the surface in comparison to the case for dry sintering. Voids are observed with a range of sizes—larger than the latex particle size, and smaller than the size of a bacterial cell. Using the relationship derived using Figure 7 with a reference sintering time of 30 min, the average void size is predicted to be approximately 4× the radius of the latex particles. Analysis of the images finds that the average void size is 4.8 ± 0.7× the particle radius.

Figure 9d shows an example of an individual bacterium in the wet-sintered biocoating. The bacterium is constrained on all sides, with randomly packed latex particles surrounding its perimeter. There is less order in comparison to the dry-sintered microstructure.

### Effect of the Film Formation Method on Cell Viability

Having established that biocoatings formed by the wet sintering by immersion method have a high porosity, we next investigate how the methods and microstructures influence the bacterial viability. Specifically, we consider the effects of the type of sintering, the choice of liquid when wet sintering, and the concentration of the coagulant salt.

We postulated that the method of film formation would have a noticeable effect on the viability of the bacteria, owing to the differences in the permeability of the coatings and the osmotic and desiccation stresses imposed on the bacteria. The desiccation stress introduced by the dry sintering method, in which the water is removed from the coating, was hypothesized to reduce the viability of bacterial cells, in comparison to the method of wet sintering by immersion, which keeps the cells in liquid throughout film formation. Additionally, for comparison, we introduced the method of “moist sintering”, in which colloidal particles in a humid atmosphere are deformed under the action of the capillary pressure from condensed water under high humidity.

In a rigorous study of moist sintering, Lin and Meier^39^ used the Kelvin–Laplace equation to relate the relative humidity of water to the radius of the water meniscus between particles. They found for their experimental system in a water-saturated atmosphere, there was an equilibrium condensed water content of 5 vol % in a layer of packed colloidal particles. Experimentally, they provided evidence that the capillary pressure from this condensed water is large enough to deform latex particles.

In our experiments, after coagulant dipping, coatings were placed for 2 h under a high relative humidity (RH) of 85% in a sealed container within an oven (Heratherm, Thermo Scientific) set to 34 °C. Then the coatings were stored at room temperature (21 °C) and 40% RH in a humidity chamber prior to analysis. Our dynamic vapor sorption gravimetric analysis found that, at 85% RH, colloidal layers contained more than 1 wt % equilibrium water content (see Figure S11 and Table S2 in the Supporting Information).

All three methods of film formation (dry, moist and wet sintering) began with the same experimental steps. For each, the coagulant dipping process used a paper substrate coated with Ca­(NO_3_)_2_·4­(H_2_O) with an areal density of 0.011 mol/m^2^. Therefore, each of the samples initially contained approximately the same number of bacterial cells. The difference in the film formation method came after the gel-coating stage. We hypothesized that reduced osmotic and desiccation stresses and greater permeability from wet sintering would lead to a higher viability of the confined cells.

Adenosine triphosphate (ATP) assays were used as a fast and sensitive technique for determining the viability of bacterial cells contained within the biocoatings.^8^ ATP is an organic nucleoside triphosphate that is present in living cells in both plant and animal matter.^58^ The ATP concentration is depleted rapidly when cells are damaged or die, thereby making the ATP concentration a good indicator of the number of living cells. The ATP concentration is positively correlated with the number of viable cells (see data from suspensions in Supporting Information). Because the pores in the biocoatings have sizes on the order of the wavelength of light (and larger), there is significant light scattering from the material. Therefore, optical microscopy techniques using cell staining assays or fluorescent protein expression to assess viability quantitatively cannot be applied reliably.

Figure 10 compares the viability, as indicated by the ATP concentration, of bacterial cells confined within coatings film-formed using one of the three different methods. A solution of PBS was used as the liquid for wet sintering in this experiment. As hypothesized, wet sintering in PBS resulted in an ATP concentration greater than a factor of 2 higher compared to the other methods. The overall *p* value between the three methods was 0.037, confirming the statistical significance of the choice of film formation on cell viability.

**Figure 10:**
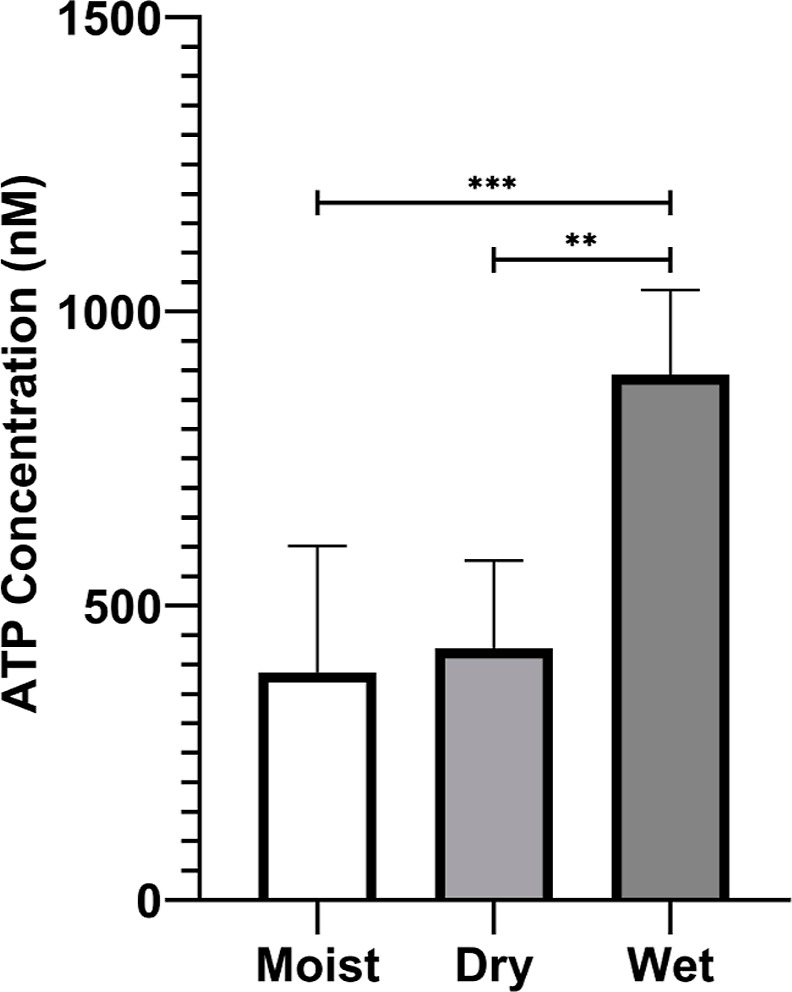
Effect of the method of film formation (moist, dry or wet sintering) on the viability of *E. coli* bacterial cells within biocoatings. PBS was used for the wet sintering method; the biocoating was not dried afterward. Each bar represents the mean of at least three biological repeats, each consisting of three technical replicates. The error bars are obtained from the standard error in the mean of the biological repeats. Each of the three methods included a sintering process at an elevated temperature of >30 °C (which is above the copolymer’s *T*_g_). The viability of bacteria in the wet sintered coatings is significantly greater than when moist sintered (*p* = 0.0005) and dry sintered (*p* = 0.0008). (*N* = 3; * *p* < 0.05; ** *p* < 0.01; *** *p* < 0.001; **** *p* < 0.0001). A Kruskal Wallis test with Dunn's multiple comparisons was used.

In the dry sintering method of film formation, the bacterial cells are exposed to air without residual water. The bacterial cells experience dehydration and an associated loss of viability. Dry sintering compared to immersion in PBS has a lower viability (*p* = 0.0008). We conclude that being in a high ionic concentration, as in the process of moist sintering, is more damaging to cell viability than allowing the coagulant salt to dry in the coating in the process of dry sintering.

Both moist and dry sintering resulted in significantly lower cell viabilities. The evaporation of water will increase the concentration of the residual coagulant salt in the biocoating. Using typical experimental parameters in Routh’s analytical model,^47^ the ionic concentration is estimated to increase by an order of magnitude when the volume fraction of colloid particles reaches 0.6 (random packing). The concentration of Ca­(NO_3_)_2_ in solution will continue to increase up to its saturation point of 5.5 M.^40^ High concentrations of salt solution can be toxic to bacterial cells.^59^ The increase in salt concentration during film formation could lead to fatal damage to the cell membrane, proteins, and DNA.^60^

With moist sintering at 85% RH, approximately 1 wt % equilibrium condensed water is retained in the colloidal film, according to our gravimetric analysis. The presence of water surrounding the cells with a high ionic concentration will create an osmotic stress during moist sintering, which explains the lower viability. In the dry sintering method of film formation, the bacterial cells are exposed to air without residual water. The bacterial cells will experience dehydration during sintering, which explains their associated loss of viability compared to wet sintering in PBS (*p* = 0.0008).

We conclude that wet sintering by immersion in PBS, where the film never dries, is the preferred method of film formation to obtain the highest viability in biocoatings. With this method, the bacterial cells experience neither dehydration nor significant osmotic stress.

### Liquid Choice for Immersion and Effect of Drying after Wet-Sintering by Immersion

Our experiments showed a strong positive effect of wet sintering by immersion on the bacterial viability. In follow-on experiments, four different liquids were compared: deionized water, PBS, M9 growth media, and lysogeny broth (LB). When the biocoatings were stored in liquid after wet sintering, it was found that immersion in LB yielded the highest viability, with the ATP concentration being approximately 2 orders of magnitude greater than immersion in water, see Figure 11 (*p* = 0.0003).

**Figure 11:**
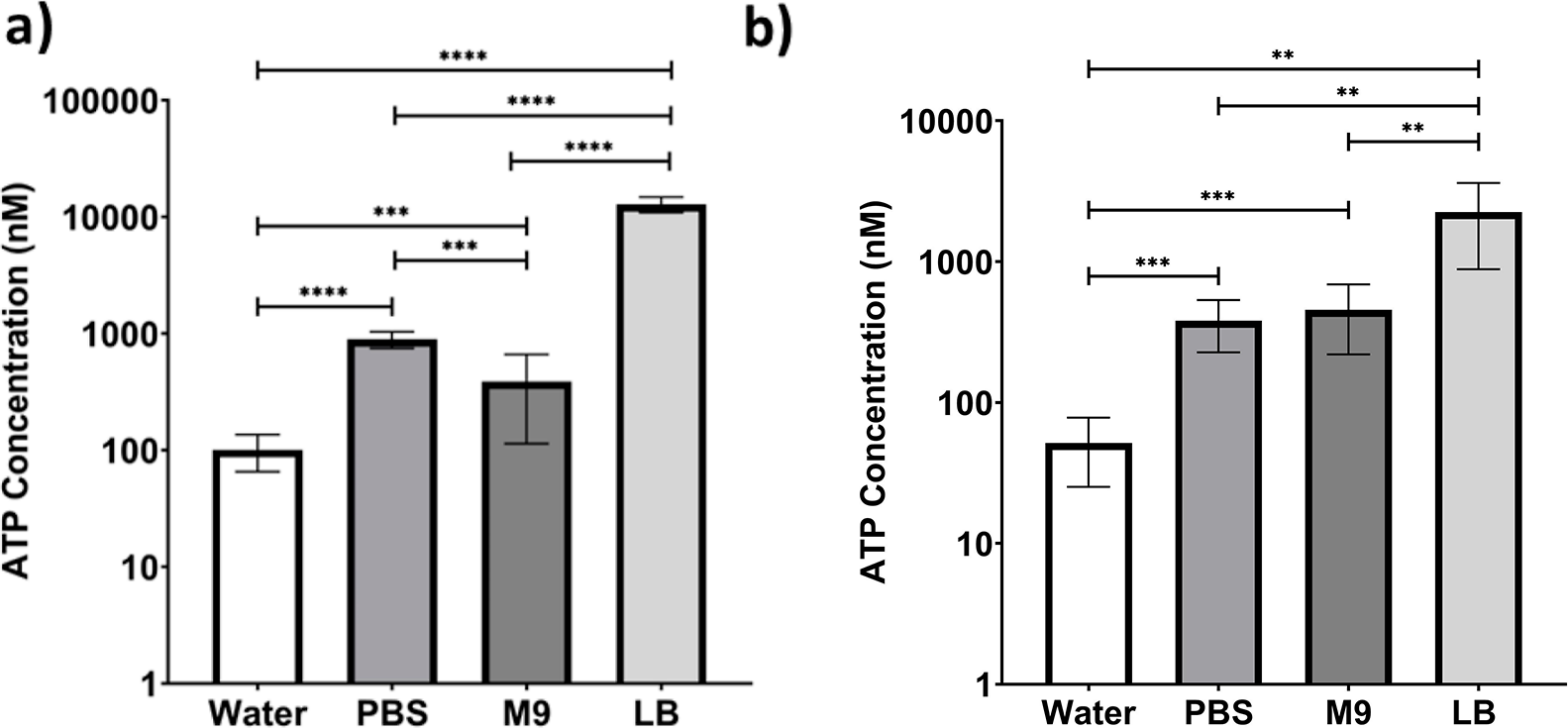
Effect of the liquid for immersion on the viability of *E. coli* bacterial cells within biocoatings. Two different wet sintering methods are compared: (a) storing the biocoatings in the liquid after film formation by wet sintering. (b) Drying the biocoatings in air after wet sintering. Each bar represents the mean of at least three biological repeats, each consisting of three technical replicates. The error bars represent the standard error in the mean of three biological repeats. ANOVA with post hoc T-test with Welch's correction was used (in combination with Welch's correction and Mann Whitney test for (a)). (*N* = 3; ** *p* < 0.01; *** *p* < 0.001; **** *p* < 0.0001).

We suspect that wet sintering in water resulted in the lowest viability of bacteria because osmotic pressure causes cells to rupture. Immersion in PBS and M9 media both yielded similar levels of viability with no significant differences between them. These two immersion media yielded a significantly higher viability than water (PBS: *p* = 0.0012, M9 media: *p* = 0.0008), but had a lower viability than LB as a sintering medium. This is because LB contains yeast extract, which allows for bacterial growth, whereas PBS is composed of salts that maintain cell viability but do not promote growth. M9 media is not enriched (unlike LB) and yields results similar to PBS.

We also investigated the effect of drying the sample in air after wet sintering. See Figure 11b. In general, drying (followed by rehydrating to perform the ATP assay) had the effect of lowering the viability of the bacterial cells by less than an order of magnitude. We infer that drying has this negative effect on the viability of the bacterial cells because of both osmotic stress and dehydration during this process. PBS is isotonic at the original concentration used in wet sintering. However, when water is lost by evaporation, the ionic concentration will increase.

When the biocoatings that were immersed in deionized water are withdrawn from the liquid, the evaporation of the surrounding water places an additional stress on the cells. The resulting viability is very low, showing that the majority of the bacteria are dead. In this case, cells are more likely to be dehydrated because the cell membranes could be damaged by osmotic pressure from the water during wet sintering. The results show the severe combined effects of osmotic pressure and dehydration.

The effect of postsintering drying on the viability was not as significant as the choice of liquid for immersion. However, the combination of the two parameters should be taken into account to achieve optimum viability. When biocoatings were wet sintered in LB and then left immersed in the liquid, they had an approximately six times greater ATP concentration than when dried and rehydrated (*p* < 0.0001). We conclude that leaving a sample immersed in an enriched media is the better technique, as it eliminates desiccation stress and provides a continuous stream of chemical substrates to the confined bacteria.

To put our results in perspective, it is useful to compare the ATP assay for the coatings wet sintered in LB and kept immersed with that for the dry sintered coatings. The ATP concentrations can be converted to the concentration of viable cells, measured as colony-forming units (CFU) per mL, assuming that the luminescence from biocoatings and cell suspensions is equivalent (see Figures S12 and S13 in the Supporting Information). This calculation allows a comparison of the viable cell densities in the biocoatings. Film formation by wet sintering in LB yields a CFU/mL value that is approximately 500 times greater than obtained with dry sintering. This significant increase in viability will drive the greater future use of biocoatings in applications where higher yields are needed.

### Effect of Coagulant Concentration

To achieve a high rate of reactivity from biocoatings, a high cell density (number of cells per unit area) is required. An obvious way to increase this density is to make thicker coatings. Therefore, the concentration of the coagulant salt was increased in some experiments.

In all experiments, 20 μL of Ca­(NO_3_)_2_·4­(H_2_O) aqueous solution was deposited over an area of 0.5 cm × 3.5 cm on a paper substrate. Two different concentrations of Ca­(NO_3_)_2_·4­(H_2_O) as the coagulant salt were compared: 0.2 M (leading to an areal density of the salt of 0.011 mol/m^2^) and 1 M (areal density of 0.055 mol/m^2^). The thicknesses of the resulting biocoatings (after film formation) increased by more than a factor of 3 when the coagulant coverage was raised. Using the known concentrations of the suspension of bacteria and polymer colloids, and assuming that the proportion of the two components remains the same in the biocoating, we obtained estimates of the total cell loading (including nonviable cells). See Table 1. Raising the coagulant concentration increases the cell loading to 3 ± 1 × 10^11^ CFU/m^2^.

**Table 1:** Estimated Coating Thickness and CFU/Area Obtained for Two Different Ca­(NO_3_)_2_ Coagulant Concentrations

Ca(NO_3_)_2_ concentration (M)	areal density of salt (mol/m^2^)	estimated dry coating thickness (μm)	estimated cell density (10^11^ CFU/m^2^)
0.2	0.011	30 ± 10	0.8 ± 0.2
1	0.055	100 ± 10	3 ± 1

Figure 12 shows that a lower coagulant salt concentration (0.2 M) resulted in a significantly higher viability in both the wet sintering (*p* = 0.0002) and also the moist sintering (*p* = 0.0486) methods. Increasing the coagulant concentration by a factor of 5 resulted in a decrease in the ATP concentration by an order of magnitude. The higher salt concentration led to a greater coating thickness and presumably increased the total number of cells in the biocoatings, but a very low fraction of those cells were viable.

**Figure 12:**
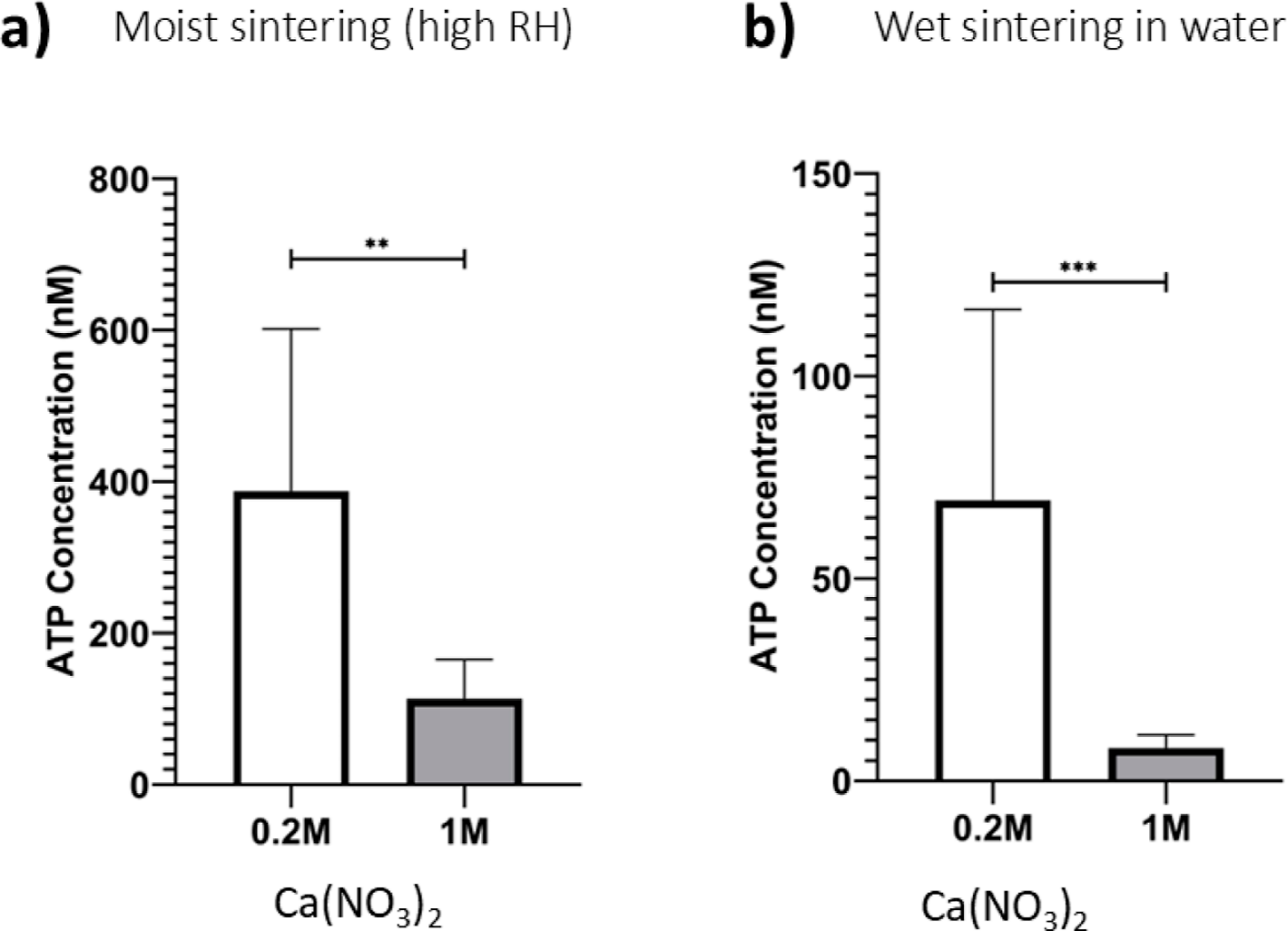
Effect of the coagulant concentration on the viability of *E. coli* cells (as determined via ATP assays) within the biocoatings. 20 μL of Ca­(NO_3_)_2_·4­(H_2_O) of known concentration (0.2 and 1 M) was deposited over an area of 0.5 cm × 3.5 cm. Two film formation methods are compared: (a) moist sintering; (b) wet sintering by immersion in water. The 1 M coagulant concentration resulted in a signficantly lower viability (as determined from the ATP assay) compared to the 0.2 M concentration when moist sintering (*p* = 0.049) and when wet sintering (*p* = 0.0002). Each bar represents the mean of at least three biological repeats, each consisting of three technical replicates. The error bars represent the standard error in the mean of the biological repeats. A T-test with Welch's correction was used. (*N* = 3; * *p* < 0.05; ** *p* < 0.01; *** *p* < 0.001; **** *p* < 0.0001).

In moist sintering, the salt is retained in the coating, whereas in wet sintering, some of the salt could diffuse out into the liquid (water) during the immersion process, thereby reducing the local salt concentration around the bacteria. However, in both cases, a higher coagulant concentration resulted in lower numbers of viable cells, despite a greater thickness of biocoating. Of course, salt on the substrate is essential for the coagulant dipping process. Therefore, an optimized amount of salt is needed to achieve the greatest counts of viable cells per area of coating.

### Metabolic Activity of Bacteria in Biocoatings and Ethanol Production

There is continued interest in using *E. coli* to generate liquid biofuels from nonpetroleum carbon sources, with genetic modification being used to increase yields.^61^ In follow-up experiments, the production of ethanol through the fermentation of glucose was used as a demonstration of the bacteria’s metabolic activity in biocoatings, while also pointing to a future application. Two biocoatings were compared. One was made via coagulation and wet sintering by immersion, whereas the other biocoating was made by the dry sintering method. Both biocoatings were found to yield ethanol when in a medium supplemented with glucose (see methods in the Supporting Information). The yield from the wet-sintered biocoating appears to be higher than the dry-sintered case (Figure S14).

## Conclusions

We have devised a new strategy for making biocoatings, which results in greater cell viability according to ATP assays. Coagulant gelation creates a porous structure of loosely packed colloidal particles. The particles are partially coalesced via a wet sintering mechanism by immersion in a suitable medium at a temperature above the polymer’s *T*_g_ to create a hard yet permeable coating. The voids in the coating undergo coarsening during wet sintering, which provides a simple means to tailor the pore size distribution and hence the permeability of the coating. When LB media was used for the wet sintering of biocoatings, *E. coli* had an estimated viable cell count that was more than 350 times greater than obtained by a conventional dry sintering process. For the greatest bacterial viability, the coagulant salt coverage on the substrate should be kept as low as possible for the desired thickness. Wet sintering of biocoatings should use LB or similar media (to avoid osmotic stress), and the biocoatings should not be dried after film formation.

These innovative methods of film formation will encourage the greater use of biocoatings for applications including environmental remediation, carbon capture, biofuel production, and biosensing, among others. In this work, the production of bioethanol via the fermentation of glucose was demonstrated in a wet-sintered biocoating. In our future work, *E. coli* confined in optimized biocoatings could be used to produce hydrogen gas via a dark fermentation process.^62,63^ The processing methods of coagulant gelation and wet sintering are applicable to any bacterial species but will be of particular benefit for desiccation-intolerant bacteria. In future work, rather than using extremophiles for carbon fixation and oxygen production in biocoatings,^8^ other common species could be used, even if they are not desiccation tolerant.

## Experimental Section

### Chemicals for Latex Synthesis

*n*-Butyl acrylate, ≥99%, containing 10–60 ppm monomethyl ether hydroquinone as inhibitor, and methyl methacrylate (MMA), 99%, containing ≥30 ppm MEHQ as inhibitor were purchased from Sigma-Aldrich. *n*-Butyl acrylate and methyl methacrylate were filtered through a short column of basic activated aluminum oxide to remove inhibitors prior to use. Acrylic acid, 98%, extra pure and stabilized was purchased from Acros Organics and filtered through basic activated aluminum oxide (Fluka). Dowfax 2A1, 46% active ingredient, was donated by the Dow Chemical Company. 2,2′-Azobis­[2-methyl-*N*-(2-hydroxyethyl)­propionamide] (VA-086) was supplied by Wako Chemicals GmbH and was recrystallized from methanol prior to use.

### Latex Synthesis

A latex formulation was developed to use the lowest possible amount of surfactant while still having colloidal stability. AA was used to impart charge stabilization. Two batches of the acrylic copolymer latex were synthesized by emulsion polymerization. The first batch was used for studies of wet sintering by immersion, and the second batch was used only for the manufacture of biocoatings.

The synthesis of the second batch, described here, was made by scaling up the first batch by a factor of 3. Dowfax 2A1 surfactant (1.24 g) was diluted with an equal mass of deionized water to make a solution. An aqueous solution of VA-086 initiator (0.67 g) was prepared in 45.44 g of deionized water. A mixture of monomers was prepared from MMA (54.50 g), BA (38.53 g) and AA (0.97 g). The entire surfactant solution and the monomer mixture (102 mL) were added to a 1 L double-walled glass reactor containing 636.45 g of deionized water. The reactor was equipped with an external circulating heating bath, a Teflon anchor stirrer fitted approximately 2 cm from the bottom of the vessel, a condenser, and a temperature probe. The reactor and initiator solution were degassed for 60 min while stirring at 200 rpm. The reactor was then heated to 85 °C and a shot of the initiator solution (45 mL) was added to begin the polymerization. The total reaction time was 3 h. Samples were taken throughout these reactions to monitor the conversion via gravimetry and the particle size via dynamic light scattering. The latex was collected by hot filtering through 200 μm mesh as the reactor was drained into a sterile container. The dispersion was not buffered. The final pH was 5.

The characteristics of the two batches of latex are presented in Table 2. The critical coagulation concentration, C* of the latex for a divalent cation (Mg^2+^) was measured to be 0.07 M. Descriptions of the latex characterization methods are presented in the Supporting Information.

**Table 2:** Characteristics of the Latex from the Two Batches

	synthesis	
characteristic	batch 1	batch 2
Z-average particle diameter (nm) (*N* = 3)	144 ± 1.5 (s.d.)	140 ± 2.3 (s.d.)
particle dispersity index (%)	14.9 ± 4.6 (s.d.)	4.6 ± 2.9 (s.d.)
monomer conversion (wt %)	100 ± 1	97 ± 1
solids content (wt %)	14.2 ± 0.5	12.5 ± 0.5
midpoint glass transition temperature (°C)	28 ± 1	27 ± 1
zeta potential (mV) (*N* = 3)	–43 ± 2 (s.d.)	–31 ± 2 (s.d.)

### Substrates for Coagulant Dipping

Filter paper (Grade 5, Whatman) with a thickness of 200 μm and a pore size of 2.5 μm was used as the substrate material. This substrate was chosen because it is water absorbent and mechanically durable. Calcium nitrate tetrahydrate (Ca­(NO_3_)_2_·4H_2_O, 99%, Sigma-Aldrich) was selected as the coagulant because it has good coatability, hygroscopicity, and tendency not to form large crystals. A divalent cation is preferred because of its lower coagulation concentration compared to monovalent cations as per the Schulze–Hardy rule.^64^ 20 μL of a Ca­(NO_3_)_2_·4­(H_2_O) solution with a concentration of 0.2 or 1 M solution (depending on the experiment) was deposited by dropping over an area of 0.5 cm × 3.5 cm of filter paper substrate on a hot plate set to 80 °C. After drying for >15 min the substrate was then left to equilibrate at room temperature (21 °C).

The areal density of the coagulant, ρ_c_ was obtained via eq 1 using the known values of coagulant salt concentration, *c*, the deposited volume of the coagulant, *V*_c_, and the surface area of substrate, *A*_s_

1 $$\rho_{c}=\frac{cV_{c}}{A_{s}}$$

A dwell time of 20 s and areal density of 0.057 mol/m^2^ of calcium nitrate tetra-hydrate were found to give a uniform coating with a thickness of >200 μm. This areal density was obtained using a 1 M solution of calcium nitrate tetra-hydrate (4.723 g of calcium nitrate tetra-hydrate added to 20 mL of deionized water). A volume of 20 μL of this solution was dropped over an area of 3.5 cm^2^ to obtain an average areal density of 0.057 mol/m^2^. This areal density was used for the majority of experiments.

### Bacteria Culturing

Nonpathogenic *E. coli* BW25113, which is the lab-adapted parent strain of the Keio collection^65^ was used in the experiments as a model organism.

Lysogeny broth (LB) was prepared from powdered form by following the manufacturer’s recommended method of dissolving 25 g/L in deionized water and adjusting the pH to 7.2 before autoclaving at 121 °C for 15 min to sterilize the solution. The LB was composed of 10 g/L casein digest peptone, 10 g/L sodium chloride, and 5 g/L yeast extract. The presence of the yeast extract allows cell growth.

Phosphate-buffered saline (PBS, Oxoid) solutions were prepared from tablet form by adding one tablet to 100 mL of deionized water. The solution was composed of 8 g/L sodium chloride, 0.2 g/L potassium chloride, 1.15 g/L disodium hydrogen phosphate, and 0.2 g/L potassium dihydrogen phosphate. The PBS was sterilized by autoclaving at 115 °C for 10 min, as per the manufacturer’s recommendation.

A 50 mL bacterial culture in LB was prepared with a starting optical density (OD) at a wavelength of 600 nm of 0.05. When the bacteria grew to an OD of 0.8–1.4 (approximately 10^10^ CFU/mL where CFU is a colony-forming unit), 50 mL was centrifuged for 10 min at 5000*g*, and the supernatant was removed. The bacterial pellet was resuspended in 1 mL LB.

For some wet sintering experiments, M9 growth media (Sigma-Aldrich) was prepared by dissolving 10.5 g of the starting powder in deionized water, adjusting the pH, and sterilizing by autoclaving at 121 °C for 15 min. 1 M magnesium sulfate (MgSO_4_) and a 20% dispersion of glucose were prepared separately and sterilized by filtration through a filter with a pore size of 0.22 μm. When the M9 has cooled, 2 mL of 1 M MgSO_4_ and 20 mL of the glucose dispersion were added per liter of M9, as per the manufacturer’s recommendation. The final solution contained salts with concentrations of 6 g/L disodium hydrogen, 3 g/L potassium dihydrogen phosphate, 0.5 g/L sodium chloride, and 1 g/L ammonium chloride. Glucose is a carbon source for the bacteria.

### Film Formation for Varying Times and Temperatures

A motorized dipping apparatus (texture analyzer, TA-XT Plus, Stable Micro Systems, Godalming, UK) was used to control the coagulant gelation process. The salt-coated substrates were dipped vertically into the latex suspension for a set dwell time (typically 20 s), and withdrawn at a speed of 10 mm/s in the vertical direction.

After coagulant gelation, the coatings underwent film formation via wet sintering. For wet sintering, the coatings were directly immersed in water (or another liquid) by affixing to the top of a beaker with adhesive putty. Samples were held in the liquid for a fixed period of time at a fixed elevated temperature when placed on a hot plate (Fisher Scientific, Isotemp). A range of water temperatures (23 °C, 29 °C, 35 °C, 38 °C, 45 °C, 49 and 59 °C) was used in wet sintering experiments. In other experiments, the temperature was fixed and the sample was removed after a range of wet sintering times.

After wet sintering, the coated substrates were taken out of the water (or liquid), and the excess water was drained off. The coated substrates were placed in a humidity chamber at 85% RH (set by a saturated solution of potassium chloride) at a temperature of 23 °C to retain hydration. The thicknesses of the substrate and dry coatings were measured using Vernier calipers.

### Film Formation of Biocoatings

A mixture was prepared by adding 1.5 mL of *E. coli* BW25113 suspension to 10 mL of the latex dispersion. Coatings were film-formed using wet, moist or dry sintering. The method of coagulant dipping and wet sintering as previously described for the standard coatings was followed. Wet sintering was conducted in deionized water, PBS, LB or M9 growth media aerobically in the laboratory atmosphere. In some experiments, the wet-sintered coatings were stored in the liquid at room temperature (rather than being stored in a humid atmosphere).

For moist sintering, the RH was maintained at ∼85% by placing the samples into sealed chambers containing saturated solutions of KCl. For dry sintering, the gel coatings were suspended in a temperature-controlled room at 30 °C and an RH of 40% for 2 h before being returned to a temperature of approximately 21 °C.

### ATP Assays

ATP generates light when using the enzyme luciferase as a catalyst. The measured light intensity is linearly related to the ATP concentration.^66^ A master curve to relate the luminescence light intensity to the ATP concentration was taken from the literature.^8^ See Figure S12 in the Supporting Information.

Biocoatings on paper were cut into three 0.5 cm × 0.5 cm squares, placed into white 96-well plates (Thermo Scientific Sterilin, 611F96WT) and 100 μL of PBS (Thermo Fisher) was added to each well.

To perform the ATP assays, 100 μL of CellTiter-Glo 3D Cell Viability Assay (Promega, G9681, Madison, WI, USA) was added and left for 30 min at room temperature. The luminescence was read on a CLARIOstar Plus (BMG LABTECH, Aylesbury, UK) and converted to ATP concentration using a master curve (Figure S12 in Supporting Information).

The three measurements taken from different regions of the same dipped sample are referred to as “technical repeats” and were averaged to give one mean data value. For each type of sample, at least three “biological repeats” were obtained by replicating the experiment on different days, with a new batch of the latex/bacteria mixture. The mean and associated standard deviations from the biological repeats (typically *N* = 3) were obtained.

The *p*-values were obtained using GraphPad Prism software (version 8.4.3 for Windows, San Diego, USA) to provide a numerical representation of the statistical significance between samples. The smaller the *p* value, the greater the statistical significance between tests. Generally, the 5% significance level (corresponding to *p* values < 0.05) was used as an indicator of significance.

## Supplementary Material



## Notes

Descriptions of latex characterization methods (dynamic light scattering, differential scanning calorimetry, gravimetry, critical coagulation concentration); coatings characterization methods (scanning electron microscopy (SEM), liquid water permeability, pendulum hardness); SEM images of biocoatings; water permeability data; image analysis; method for measuring film shrinkage during wet sintering; dynamic vapor sorption data; ATP assay master curves (ATP concentration, luminescence, and CFU/mL relationships) (PDF)
