# Supplementary material for: Biocoatings with Enhanced Bacterial Viability via Coagulant Dipping and Wet Sintering by Immersion
Source: ACS Appl Mater Interfaces. 2026 Apr 17;18(16):23693–707. doi: 10.1021/acsami.6c05652 (PMC13133788; doi:10.1021/acsami.6c05652)
Supplement: Supplementary file 1 [file am6c05652_si_001.pdf]

SUPPORTING INFORMATION
Biocoatings with Enhanced Bacterial Viability via Coagulant Dipping and Wet Sintering by Immersion

Alexia M. J. M. Beale,† Kathleen L. Dunbar,† Emily M. Brogden,‡ Solomon S. Melides,† Richard P. Sear,† Stefan A. F. Bon,‡ Suzanne M. Hingley-Wilson,¶ and Joseph L. Keddie*,†

†*School of Mathematics and Physics, University of Surrey, Guildford, Surrey, GU2 7XH, United Kingdom*

‡*Department of Chemistry, University of Warwick, Coventry CV4 7AL, United Kingdom*

¶*Discipline of Microbiology, Infection and Immunity, University of Surrey, Guildford, Surrey, GU2 7XH, United Kingdom*

E-mail: j.keddie@surrey.ac.uk

Latex Characterization Methods

Dynamic light scattering (DLS)

The average hydrodynamic particle diameter and dispersity index were recorded using an Anton Paar Litesizer 500 (0.3 - 2,000 nm). A sample of latex (5 mg) was diluted with distilled water (7 ml). A disposable cuvette was rinsed with distilled water filtered through a 0.25 μm hydrophilic PTFE syringe filter. A disposable cuvette was half filled with the di-

lute latex sample and three size measurements were recorded (backscatter 175°, a 4-minute equilibration time at 25 $^{\circ}C$, a refractive index for water of 1.3303) with the average hydrodynamic diameter quoted being an average of the three measurements. An Omega cuvette Mat. No. 225288 was filled with the same diluted latex solution and three zeta potential measurements were recorded (Smoluchowski approximation, a 4 min. equilibration time at 25 $^{\circ}C$, a refractive index for water of 1.3303) with the average zeta potential quoted being an average of three measurements.

Differential scanning calorimetry (DSC)

DSC measurements were carried out on a Metler Toledo STARe instrument, using a 40 μL aluminum DSC flat pan with an aluminium lid. Approximately 10 mg of a latex sample was dried in the pan, and then it was sealed with the lid. Heat-cool-heat cycles were carried out at a rate of 10 $Kmin^{-1}$. The glass transition temperatures were determined using the midpoint at half height from the second heating ramp.

Gravimetry for Monomer Conversion Measurements

Approximately 0.5 ml of sample was removed from the reactor, using a degassed syringe, for gravimetric analysis. The weight of the aluminum gravimetry pan (P) was measured. The sample was then syringed into the pan immediately after removing from the reactor. The mass of the pan when containing the wet sample was recorded. The sample was left to dry in a fumehood overnight, and then dried at 105 $^{\circ}C$ in a vacuum oven overnight. The dry mass of the sample and pan (P_d) was then recorded. This value was used to calculate the solids content, SC, at each time point using Equation 1, where P_w is the mass of the wet pan.

$$SC = \frac{P_d - P}{P_w - P} \tag{1}$$

The SC was then used to calculate the conversion, X_m at each time point using Equation

2, where M_{sol} is the mass of all solid components, not including polymer, M_{tot} is the mass of all components, and M_{mon} is the total mass of monomer.

$$X_m = (SC - \frac{M_{solid}}{M_{total}}) \times (\frac{M_{total}}{M_{mon}}) \tag{2}$$

Critical Coagulation Concentration (CCC)

Coagulant solutions of $MgCl_2$ were prepared using Type 2 deionized water. The CCC was estimated by preparing $MgCl_2$ solutions at concentrations ranging between 0.005 M and 1 M. Then, 200 μl of the latex dispersion was added to each of the $MgCl_2$ solutions. Coagulation was determined visually by observing the formation of visible coagulants and sedimentation after a few minutes. At $MgCl_2$ concentrations of 0.07 M and higher, sedimentation was apparent. However, the latex was stable at a concentration of 0.06 M.

Coatings Characterization Methods

Coating Method

Figure S1 illustrates the experimental process of forming a gel coating followed by wet sintering by immersion in water.

Scanning Electron Microscopy (SEM)

For SEM imaging, a 0.5 cm × 0.5 cm region was cut from the sample and attached to a metal stub using carbon tape. The samples were coated with two 3 nm layers of gold using a gold coater (Q 150 V ES Plus, Quorum, UK) and silver DAG was applied to the corners to minimize charging effects. Microstructures were imaged using a scanning electron microscope (Apreo, ThermoFisher, UK) with an accelerating voltage of 1 kV and a beam current of 6.3 pA.

In complementary analysis, a Canon Ixus camera was used to image samples for observation of the macrostructure and to characterise the thickness of wet gel films.

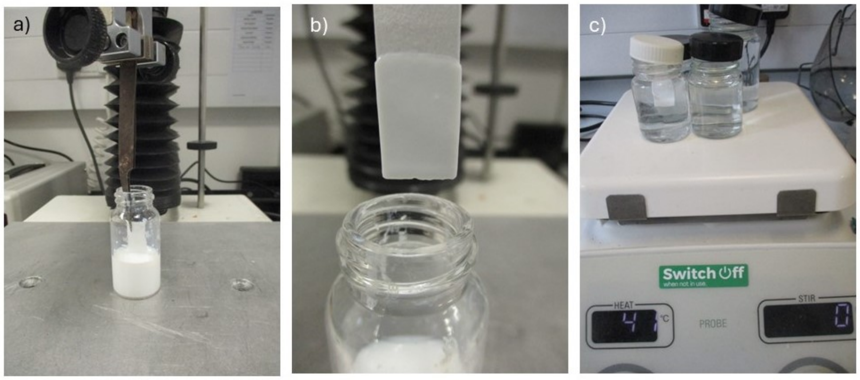

Figure S1: The coagulant dipping and wet sintering by immersion method. a) The filter paper substrate was dipped into the latex suspension for a known dip time using a motorised dipping apparatus. b) An example gel when withdrawn from the latex suspension. c) Wet sintering by immersion: Samples suspended using adhesive putty to adhere them to the interior edge of the rim of a glass beaker, immersed in water at an elevated temperature obtained by heating using a hot plate.

SEM Cross-Sections

To obtain the cross-sectional SEM images, the coated samples were first frozen by immersion into liquid nitrogen, and then immediately cut using a razor blade. The samples were then adhered to cross-sectional aluminum stubs using carbon tape and the rest of the method described above for surface SEM image analysis was followed.

Liquid Water Permeability

Filter paper with a circular area of 12 cm^2 was coated by coagulant dipping. First, 1 ml of 1 M $(CaNO_3)_2 \cdot 4H_2O$ solution was spread over this area. Then the dried coagulant-coated substrate was immersed into a latex bath and withdrawn. The coated sample was then film-formed by immersion in deionized water in an oven at 34 $°C$ for 30 min. After removing the gel-coated sample from the water, the uncoated part of the paper substrate, which had acted as a holder when immersed, was cut using a razor blade. The remaining coated sample was used for the measurement.

For comparison, a second coating was prepared from an acrylic copolymer latex with a glass transition temperature of 18 $°C$. This latex was made in a similar way to the other,

except that the MMA:BA mass ratio was reduced to 1.2:1. This coating was made in a conventional way without coagulation and with free evaporation. To ensure a thick enough coating, two layers were deposited. 1 ml of latex was dropped and spread onto the circular filter paper. After film formation in an oven set to 34 $°C$ for approximately 30 min, an additional 1 ml of latex was drop-cast once the first layer and film-formed in the same way.

The permeability of the paper substrate was also measured, to allow the analysis of the coatings' permeability. The paper substrate was cut to size (a nearly circular piece with an approximate area of 12 cm^2) and used directly. Three replicates were made for each sample.

Aluminum permeability cups with an active area of 10 cm^2 (Industrial Physics) were used to measure the permeability of latex coatings. The coated paper was placed between two rubber rings and sealed with silicone grease (RS, Stock No.:494-124). The permeability cup was filled with deionized water and inverted to allow for the measurement of the liquid. The mass of the filled cup was recorded at 10 s intervals using a digital mass balance (Entris, Sartorius). The inverted permeability cup was positioned on a stand to allow for air flow beneath it. The room's RH was recorded at the beginning and end of each set of measurements. The RH varied between 53 and 57 % and was included in the calculation of permeability. The mass loss was determined by the subtraction of the initial mass of the sample from the value at time t. The values of permeability were found from a linear fit to the mass loss data and using the standard equations presented next.[1,2]

Fick's first law of diffusion gives the flux J (kg/m^2s) of the permeants through a layer:

$$J = \frac{D\rho\Delta a}{L_T} \tag{3}$$

where D (m^2s^{-1}) is the diffusion coefficient of the permeate, Δa is the difference in activity between the two sides of the sample and L_T (m) is the sample thickness.[1]

With a multilayer film consisting of N layers of thickness L_i each with associated permeability P_i the total permeability P_T is given by

$$P_T = \frac{L_T}{\frac{L_1}{P_1} + \frac{L_2}{P_2} + \dots \frac{L_N}{P_N}} \tag{4}$$

where L_T is the total thickness of the sample (i.e. $L_T = L_1 + L_2 + ... + L_N$).

In this research, there is a focus on dip-coated samples, with both sides of the substrate coated with a single layer of polymeric gel coating, resulting in a tri-layer structure of coating/paper/coating.

In the case of these dip-coated films, the total permeability is given by

$$P_T = \frac{L_T}{\frac{L_C}{P_C} + \frac{L_S}{P_S} + \frac{L_C}{P_C}} = \frac{L_T}{\frac{2L_C}{P_C} + \frac{L_S}{P_S}} \tag{5}$$

where L_C is the thickness of the coating, L_S is the thickness of the substrate and $L_T = L_C + L_S + L_C = L_S + 2L_C$.[2]

The total permeability P_T may be measured experimentally, e.g. by using permeability cups or permeability cells, allowing for the permeability of the coating P_C to be determined by the following rearrangement of Equation 5:

$$P_C = \frac{L_T - L_S}{\frac{L_T}{P_T} - \frac{L_S}{P_S}} \tag{6}$$

This is the permeability that was found for the porous coating obtained from coagulant dipping and wet sintering.

The flux J may be measured experimentally as the rate of molar mass loss per unit area:

$$J = \frac{\Delta m}{MAt} \tag{7}$$

where $\Delta m/t$ is the mass loss per unit time measured experimentally, M is the molar mass of the permeant (in our case, this is water), and A is the exposed surface area of the sample.

The total permeability of the multilayer sample may be obtained, using Equation 3 to

derive the following equation:

$$P_T = \frac{JL_T}{\Delta a} \tag{8}$$

König Pendulum Hardness

A König pendulum hardness rocker counter (Sheen Instruments Ltd., UK) was used to measure the hardness of coatings. For one measurement, the wet latex dispersion was dropped and spread onto a clean glass substrate and film-formed in a convection oven (Heratherm, Thermo Scientific or Sanyo convection oven) set to 45 $°C$ until dry. In a second measurement, the dispersion was cast onto the glass and dried at a room temperature of 21 $°C$. To make the measurement, the pendulum was started at an initial angle of 6 degrees. The number of periods (each of 1.4 s) to reach a deflection angle of 3 degrees was counted to find the total time. At least three replicate measurements were made from each of two separate samples. The ambient temperature during the hardness measurements was either 21 $°C$ or 27 $°C$.

The measurements are presented in Table S1. The coating's hardness after film formation in the oven is approximately two times higher than when the coating was not film-formed (i.e., cast at 21$°C$, which is below the copolymer's midpoint T_g of 28$°C$). When the temperature of measurement is close to the midpoint T_g, the hardness is lower because the polymer is softened as it leaves the glassy state. For comparison, the hardness of glass was measured to be 260 s on the same apparatus.[3]

Table S1: König Pendulum Hardness at Two Temperatures. The uncertainties are the standard error in the mean.

**Film Formation Temperature ($°C$)**	**Temperature of Hardness Measurement ($°C$)**	**Pendulum Hardness (s)**
21	21	34 ± 1
45	21	73 ± 1
45	27	43 ± 1

The Need for Coagulant Gelation when Wet Sintering

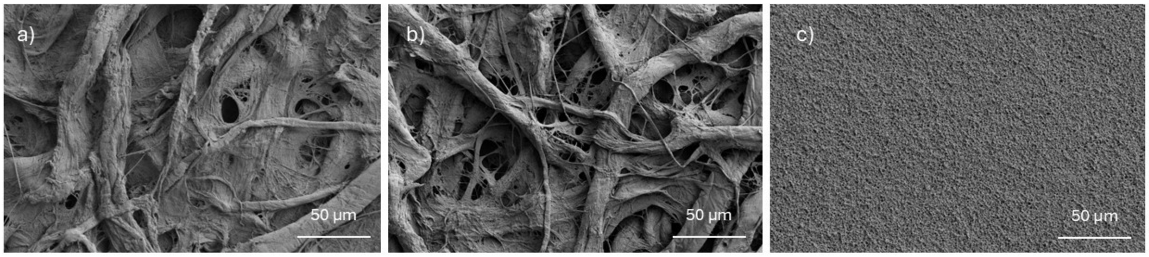

Figure S2: SEM micrographs illustrating the need for coagulant gelation to form a coating using the technique of wet sintering by immersion: a) the uncoated paper substrate, b) the resulting "coating" from the immersion method at 35 °C without a salt-coated substrate, c) an example coating formed using coagulant gelation method and wet sintering by immersion at 35 °C.

Figure S2 demonstrates the importance of coagulant gelation in the formation of coatings using the new technique of wet sintering by immersion. The micrograph of a sample formed without a coagulant (Figure b) is visually similar to the microstructure of the paper substrate (Figure a), with cellulose fibres being visible at the paper surface. No coating is apparent after the immersion step. When the paper was dipped into the dilute latex dispersion, fewer colloidal particles were adsorbed on it, and they were removed when immersed in water. Micrographs of a coating formed by the technique of coagulant gelation using wet sintering by immersion is shown for comparison (Figure c). The introduction of coagulant gelation results in a uniform coating that fully coats the paper fibers so that they are no longer visible. This comparison highlights the importance of the combination of coagulant gelation and wet sintering by immersion in the formation of coatings.

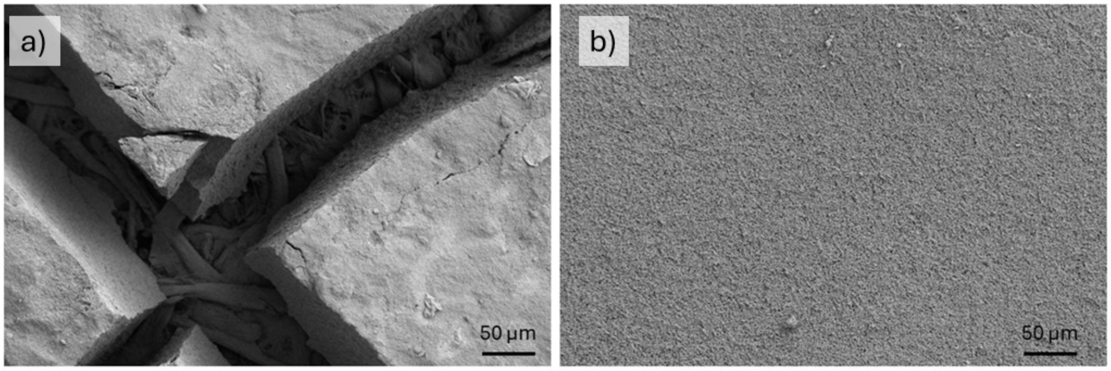

Figure S3: SEM images of coatings formed by wet sintering by immersion in de-ionised water at temperatures of a) 23 °C (which is below the polymer's T_g) and b) 35 °C (which is above the T_g).

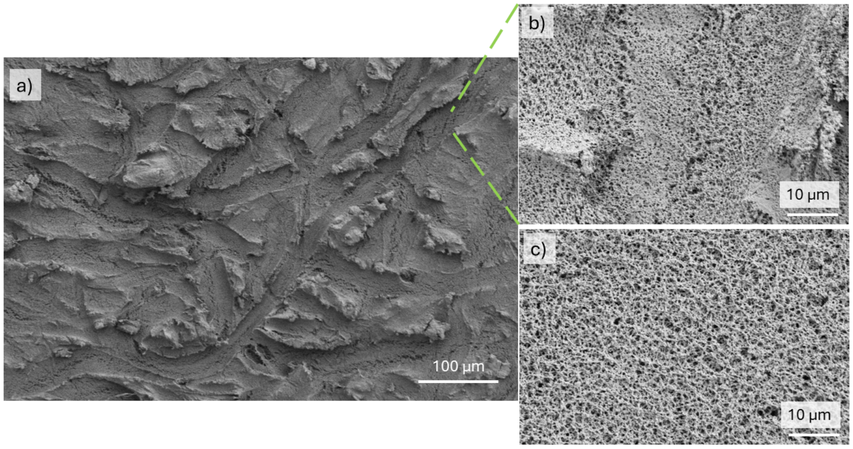

Figure S4: a) SEM micrograph of the reverse side of the coating - inserts (also obtained from SEM): b) a close-up of the structure of the reverse side of the coating; c) a corresponding image from the surface of the coating. The coating was formed by immersion in water at 35 °C for 3 hours.

The Need for the Polymer's Temperature to be Above T_g

Microstructure of the Back Side of Coagulant-Dipped, Wet Sintered Coatings

Gel Coatings on Alternative Substrates

Figure S5 shows a comparison of the microstructure of coatings obtained using the method of coagulant dipping and wet sintering by immersion on a range of different substrates: filter paper (Grade 5, Whatman), balsa wood (Hobbycraft, UK), woven cotton cloth, and

corrugated cardboard. The gel coatings were obtained by depositing 40 μl of a 1 M solution of $Ca(NO_3)_2 \cdot 4(H_2O)$ over an area of 1 cm $\times$ 3.5 cm and using the method of wet sintering by immersion in water for 30 min. at 34 $°C$.

The visual similarities between the coatings are immediately apparent. The pore size remains visually constant as the substrate is varied, showing that the method of wet sintering by immersion may be applied to different substrates.

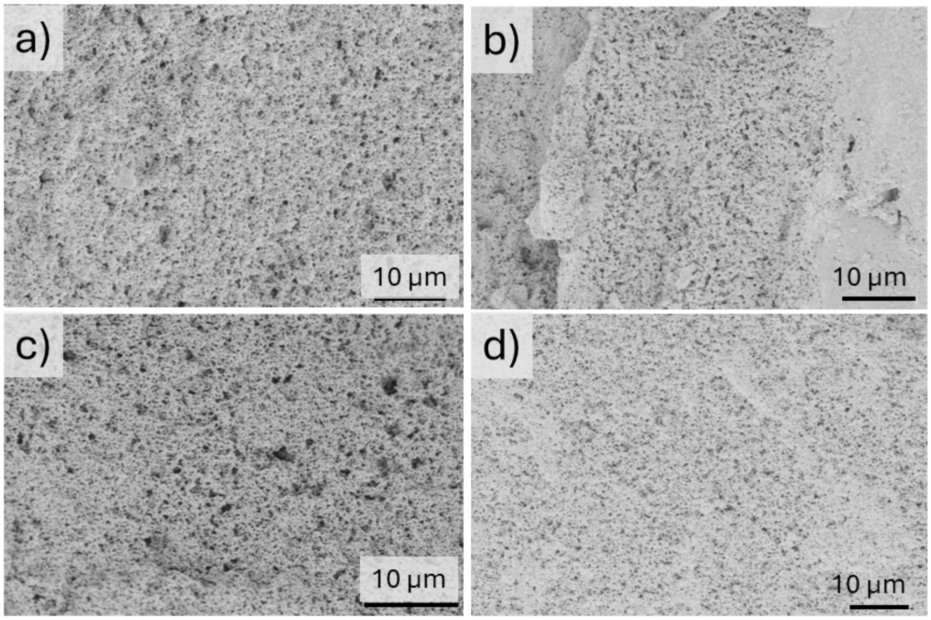

Figure S5: SEM images providing a comparison of the microstructure of the top surface gelled and wet-sintered coatings on different substrates: a) filter paper, b) balsa wood, c) cloth, d) corrugated cardboard.

The gel coating obtained using balsa wood as the substrate (Figure S5b) is less uniform than the coating obtained on the other substrates. We suggest that this is due to the non-uniformity of the surface of the substrate, and hence the non-uniformity of the coagulant coverage. Wood has grain with differing surface properties. Some regions are more absorptive and have greater adhesion to the latex gel coating than others, leading to a non-uniform gel structure.

The microstructure obtained when using a cardboard substrate (Figure S5d) also shows some areas of non-uniformity. Figure S5 also provides evidence that filter paper, which is

the substrate used in the main paper, is a good choice of substrate to obtain a uniform microstructure.

Liquid Water Permeability Results

Figure S6 shows an example raw data set for each sample type studied. All three samples show a linear decrease in mass over time, meaning that the water flux through the sample is constant. The paper substrate is seen to have a greater rate of mass loss than the two thicker, coated samples. The coagulant dipped and wet sintered sample (called T_g28 in the figure) has a greater mass loss than the non-gelled coating coalesced at a temperature above its glass transition temperature. This result is even though the gelled coating is thicker. From these raw data, the effect of the coating method on the permeability of the sample is noticeable.

Quantifying Porosity in Standard Coatings

The void size was quantified manually by identifying the four largest voids per SEM image acquired at a magnification of ×25000 by eye and using ImageJ software[4] to draw the circumference of the void and determine the area of the void. The void radius was obtained by assuming that the void was circular (using πr^2) and the average void radius from the four largest voids, repeated over two images, was measured.

To determine the percentage of pixels within pore regions, a Python script was run over 5-10 images per sample. The images were thresholded using OpenCV threshold, then cc3d connected components[5] was used to count all the thresholded pixels in clusters above a certain size (which was set to be 4 pixels to eliminate noisy pixels from the measurement). The average value from the set of images was obtained.

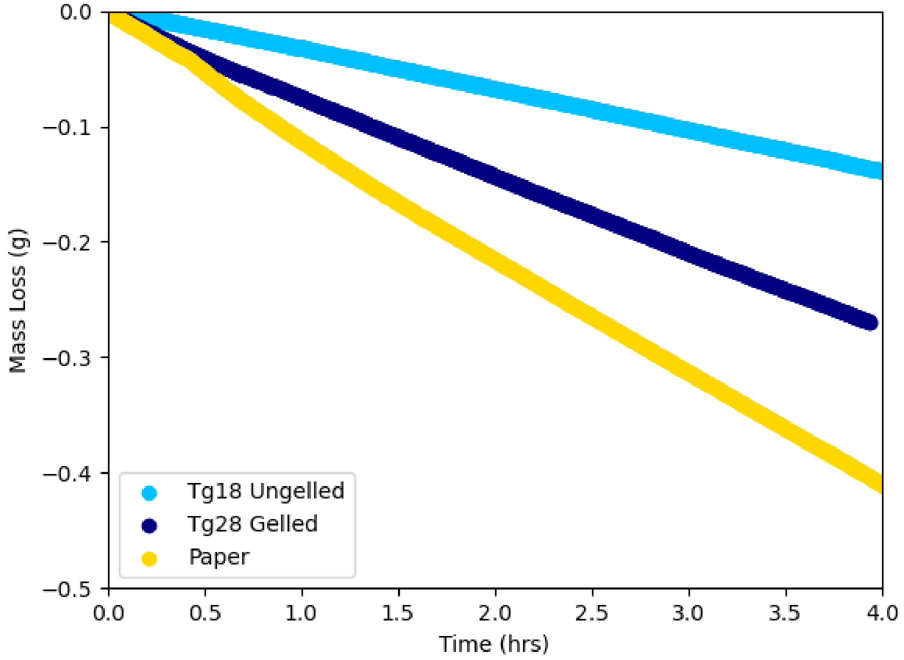

Figure S6: Comparison of the rate of mass loss from a non-gelled coating (thickness of 0.05 mm and glass transition temperature of 18 °C; called Tg18 here) and gelled coatings (coagulant dipped (0.54 mm thick) and film formed via wet sintering by immersion in water. Data from a paper substrate (thickness 0.18 mm) is shown for comparison. An example data set for each type of sample is displayed. Data from three replicates were obtained. Note that the lines consist of data points with a time interval of 10 s. The uncertainty in the mass loss is ± 0.0002 g.

Measuring the Shrinkage of the Gel Coatings

In order to investigate the shrinkage and densification of gel coatings over time during the process of wet sintering by immersion, the coated samples were suspended from a clamp stand perpendicular to a high-definition, black-and-white camera (acA1920- 155 um, Basler, Germany) fitted with a 4 × telescopic lens (Edmund Optics, NJ, US). The coated samples were immersed in water on a hotplate. (In some experiments, the samples were held vertically in air.) The width of the sample was measured by imaging from the side when illuminated by the use of an LED screen. Images were obtained at set intervals (typically 30 s) using pylon Viewer.[6] Images were calibrated by the use of an object of known width, prior to data collection.

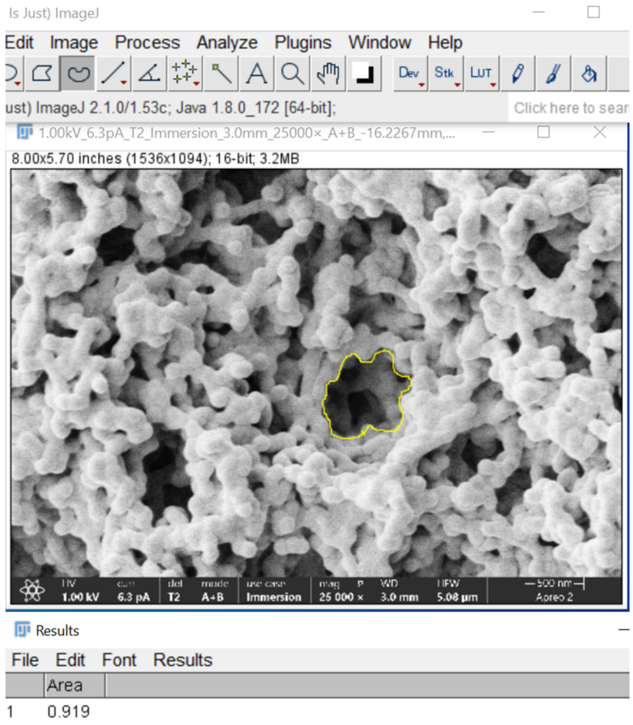

Figure S7: Using ImageJ to quantity the void size of a sample. The yellow outline shows the manually drawn perimeter of the void selected to be measured.

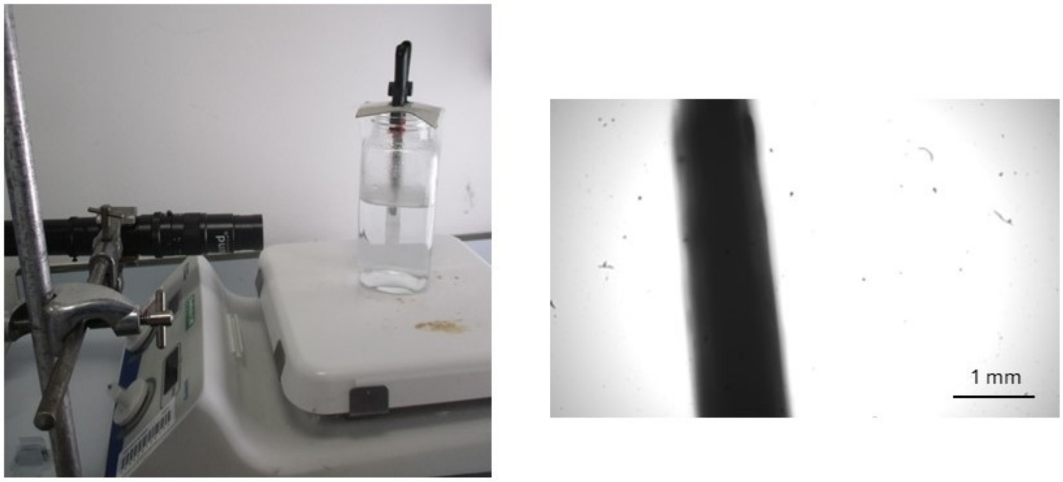

Figure S8: The experimental set-up for the measurement of the shrinkage of the gel coatings during wet sintering by immersion (LHS). A black-and-white digital camera was used to image a cross section of the gel coated paper substrate. An example image obtained is shown (RHS).

Figure S8 shows the experimental set-up to image the shrinkage of the gel coatings while immersed, with an example image shown on the right-hand side. The image shows the cross

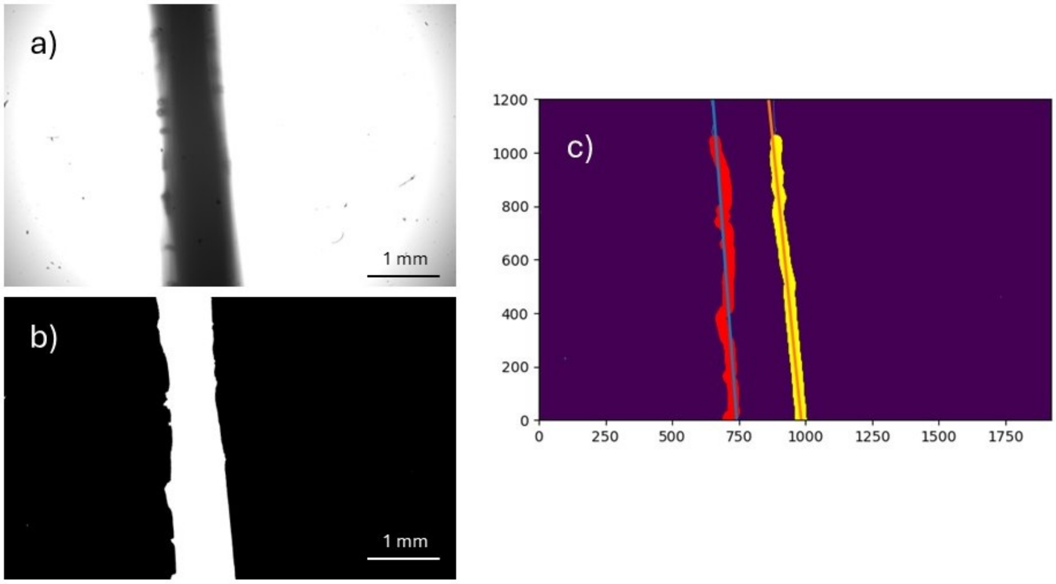

Figure S9: The shrinkage of the gel coatings during wet sintering by immersion. a) The raw image of the cross-section of the the gel-coated paper substrate (obtained using a Basler camera). b) The thresholded image. c) The detected edges of the sample are shown as the red and yellow points along with the obtained fit for the two detected edges (blue line and orange lines). The average width of the sample was obtained by the determination of the difference between the two edge detected lines.

section of the sample: a gel coated paper substrate. The average thickness of the sample was determined over time using python by looping over the time-series stack of image files. For each raw image (see Figure S9a), the edges of the paper sample's cross-section were identified by the combined use of thresholding (see Figure S9) and Canny edge detection (Figure S9c).[7] A line of best fit was applied using the method of linear regression (using the function linregress).[8,9] The average width of the sample between the two obtained edge-lines was calculated and used to form the plots shown in this section.

The thickness of the coatings as a function of time was converted to a relative density, ρ_{rel} using the mass of the dry latex coating m_{dry}. This value was determined by the subtraction

of the mass of the paper substrate from the fully dried mass of the gel-coated sample at 40% RH.

The relative density, ρ_{norm}, was determined as a function of time from the thickness of the coating, h, and its area, A using this expression:

$$\rho_{rel} = \frac{\rho}{\rho_p} = \frac{m_{dry}}{\rho_p A h} \tag{9}$$

where ρ is the density of the gel coating at a given time, and ρ_p is the density of the latex polymer (taken to be 1130 kgm^{-3}). The thickness of the gel coating, h, was obtained using digital calipers to measure the thickness of the coated sample and then subtracting the known thickness of the paper substrate.

Figure S10 shows the relative density data for a coagulant-dipped coating in air at room temperature.

As a check, the paper substrate without a coating was imaged in water. Its thickness was constant over time, which shows that swelling in water was minimal.

Analysis of Condensed Water by Dynamic Vapor Sorption Measurements

Equilibrium water vapor sorption (DVS) in the latex without film formation (not sintered) was obtained at two different relative humidities using a microbalance in controlled atmosphere in a DVS instrument (Adventure, Surface Measurement Systems, London, UK). A measurement was made at a relative humidity (RH) of 40 % to represent the ambient conditions. A second measurement was made at an RH of 85 % because that is what was used for moist sintering experiments (Figure S11).

To dry the sample, 10.2 mg of the latex dispersion was placed in a sample pan in the DVS instrument. Drying was achieved by passing 200 standard cubic centimeters (sccm) of dry nitrogen gas at a temperature of 20 $^{\circ}C$ over the pan for two days. Drying was determined to be complete when the change in mass with respect to time was less than 0.002

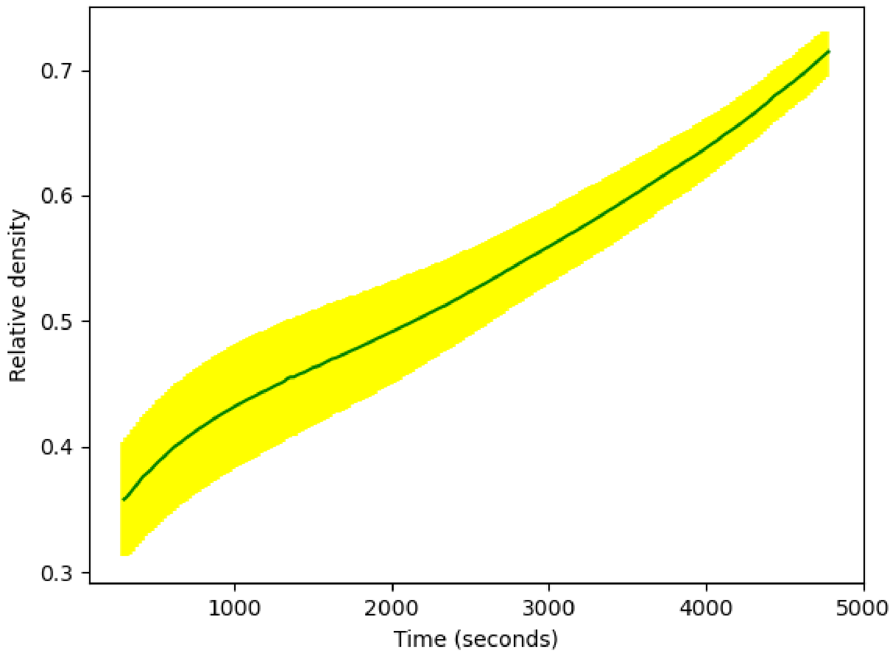

Figure S10: Relative density of gel coatings drying at room temperature (∼21°C) in air as a function of time. The yellow region displays the uncertainty in the measurements from three repeat samples.

mg/min constantly for more than 30 min. The relative humidity (RH) in the instrument was increased to 40% by passing a mixture of dry and fully hydrated nitrogen gas at a rate of 200 sccm, and the sample mass was measured over time. After the sample had reached equilibrium, the RH was raised to an RH of 85% as the increase in mass was measured until an equilibrium value was reached. The results are presented in Table S2.

Table S2: The relationship between relative humidity at 20 $°C$ and the equilibrium mass of a latex sample without film formation.

**Relative Humidity**	**Mass (mg)**	**wt.% increase in mass (compared to dried)**
0%	10.217	-
40%	10.250	0.3%
85%	10.352	1.3%

The results show that at an RH of 85% there is condensed water in the packed latex particles with a concentration of approximately 1 wt.%. At an RH of 40% (comparable to

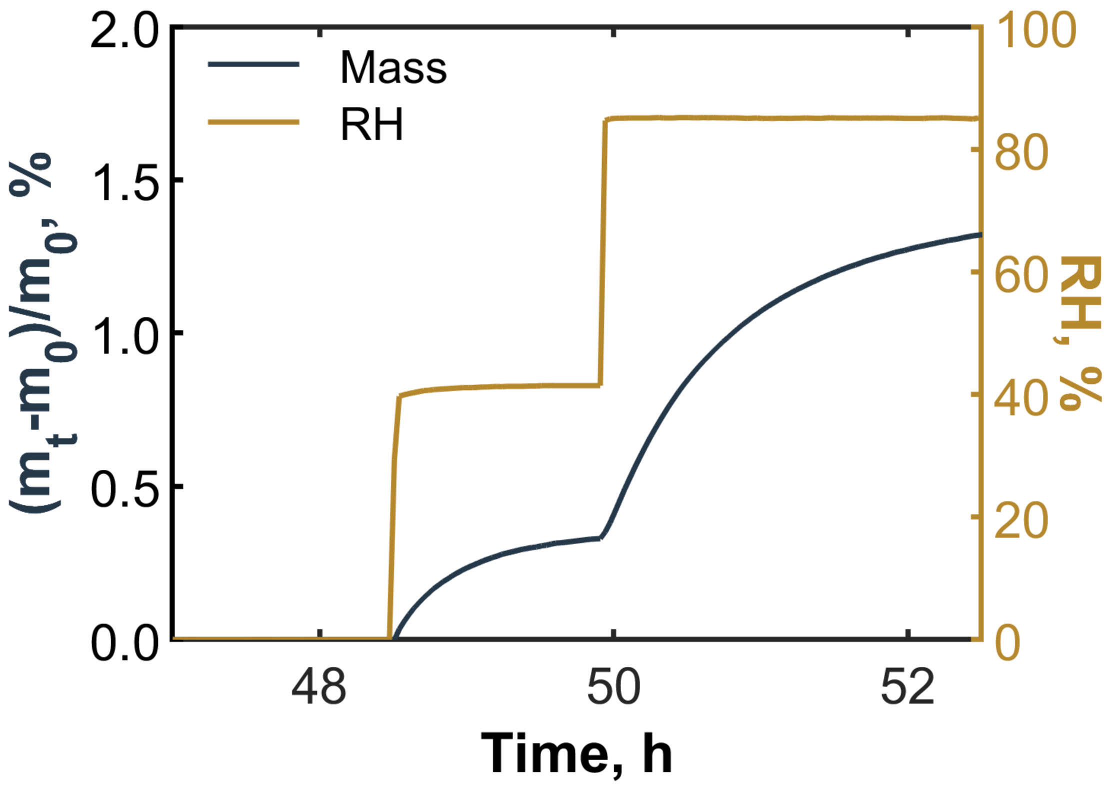

Figure S11: Dynamic vapor sorption data showing the change in mass of the latex sample (black line) when the relative humidity was increased to 40% and to 85% (gold line).

what is found in the ambient environment), there is a negligible amount of condensed water.

ATP Assay Master Curve

The luminescence was measured using the CLARIOstar Plus (BMG LABTECH, Aylesbury, UK) for known concentrations of ATP. The equation of the line of best fit to the data (Figure S12) was used to determine the ATP concentration (C_{ATP}) in units of nM from the measured values of the relative luminescence units (RLU) as:

$$\log C_{ATP} = \frac{\log{(RLU)} - 3.235}{0.6258} \tag{10}$$

The conversions from luminescence and ATP concentrations to cell counts (CFU/ml) are presented in Figure S13. Liquid cultures were diluted sequentially by a factor of five,

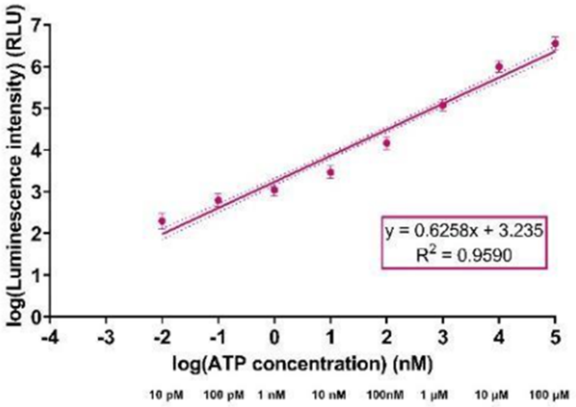

Figure S12: Dependence of logarithm of the luminescence intensity (relative luminescence units (RLU)) on $\log_{10}$ logarithm of the ATP concentration using the CellTiter-Glo 3D viability assay. The line of best fit is shown, and the equation is presented in the inset. Data taken from Krings *et al.*[3]

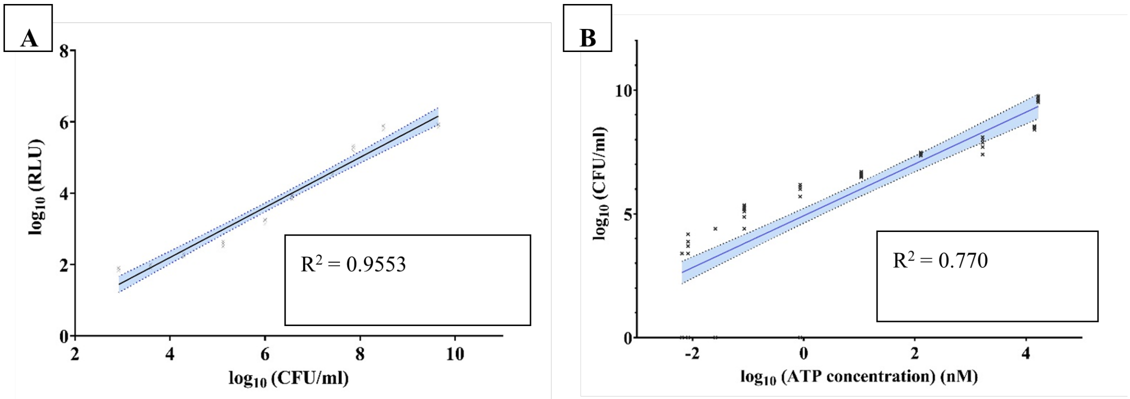

Figure S13: Standard curves for E. coli BW25113. A) E. coli concentration in units of CFU/ml as a function of the luminescence in RLU (N=3). The line of best fit is log(RLU) = 0.7013 log(CFU/ml) - 0.6071. B) ATP concentration (nM) as a function of the E. coli concentration (N=6). The line of best fit (straight line) is log (CFU/ml) =1.047 log(ATP) + 4.924.

measured using the CellTiter assay, and normalized against negative controls. The linear regressions and 95% confidence intervals are presented.

The data in Figure S13 were obtained from measurements of cells in suspension. Using the correlation of ATP concentration with cell counts (CFU/ml) as a measure of the viability in biocoatings may lead to an over-calculation of viable cell numbers. The reason is that non-growing (immobilized) bacteria are often more metabolically-active than cells in suspension, because less of their energy is expended on growth.

High-Performance Liquid Chromatography (HPLC)

Biocoatings were prepared by both dry and wet sintering, as already described for the ATP analysis. The coated paper substrates were placed in 10 mL of M9 broth supplemented with 0.5 v/v% glucose (Thermo Fisher, D-(+), anhydrous, 99%) within a Balch tube with a septum seal. An anaerobic atmosphere was created by purging with dry nitrogen gas. The biocoatings were incubated statically in the tubes at a temperature of 37 °C for 24 h.

HPLC was performed using a Vanquish Ultra-HPLC (Thermo Fisher) instrument, operating with Chromeleon software (version 7.4). The experiments used an Accucore C18 (150 mm × 2.1 mm) analytical column (Thermo Scientific). The column chamber was heated to a temperature of 25 °C using forced air. Experiments were conducted with 10 v/v % acetonitrile in water (Type 1 deionized) as the mobile phase, a run time of 5 min., a flow rate of 0.25 mL/min, and a draw speed of 3 mL/s. In each experiment, 1 mL of the medium in contact with the biocoating in the Balch tube was withdrawn through the septum and passed through a 0.22 μm filter before the HPLC analysis. A UV detector operating at 250 nm was used for all experiments. A 10 v/v% solution of ethanol in water (Type 1 deionized) was analyzed as a reference standard (and also other solution concentrations.

Results are presented in Figure S14. The 10 v/v% ethanol solution exhibited an absorbance peak centered around an elution time of 1.43 min. Analysis of the M9 medium (not shown) found no such peak in this region. The HPLC chromatograms from the wet sintered biocoating displayed a strong absorbance peak in this region (1.44 min), indicating ethanol production by the bacteria contained in it, as a result of metabolic activity. The dry sintered biocoating also yielded a peak indicative of ethanol, but with a lower height than found for the wet sintered biocoating. The ethanol concentrations obtained from the biocoatings are greater than 10 v/v% but have not been otherwise quantified.

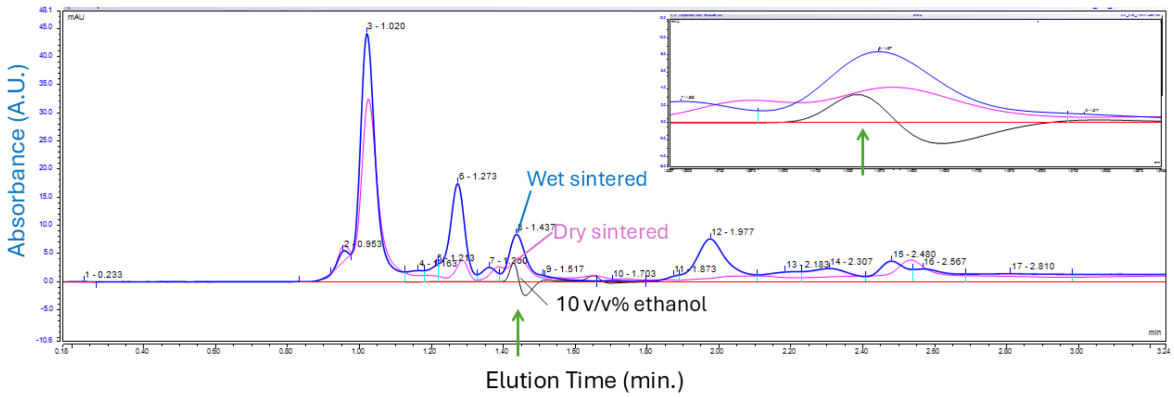

Figure S14: HPLC chromatograms obtained from a wet sintered (blue line) and a dry sintered (pink line) biocoating, in comparison to a 10 v/v % ethanol solution (black line). The absorbance peak for ethanol is centered around 1.43 min (indicated by the green arrow). The inset shows a magnified view of the region of interest for ethanol around an elution time of 1.43 min.

References

(1) Baukh, V.; Huinink, H. P.; Adan, O. C.; Erich, S. J.; van der Ven, L. G. Predicting water transport in multilayer coatings. *Polymer* **2012**, *53*, 3304–3312.

(2) Solovyov, S. E.; Goldman, A. Y. Permeability of multi-layer structures. *e-Polymers* **2004**, *4*, 023.

(3) Krings, S.; Chen, Y.; Keddie, J. L.; Hingley-Wilson, S. Oxygen evolution from extremophilic cyanobacteria confined in hard biocoatings. *Microbiology Spectrum* **2023**, *11*, e01870–23.

(4) Rasband, W. S. ImageJ. `https://imagej.net/ij/`.

(5) Anaconda. 2025; `https://anaconda.org/conda-forge/connected-components-3d`.

(6) Basler. 2025; `https://www.baslerweb.com/en/software/pylon/pylon-viewer/`.

(7) Bradski, G. The OpenCV Library. *Dr. Dobb's Journal of Software Tools* **2000**,

(8) SciPy. 2025; `https://docs.scipy.org/doc/scipy/reference/generated/scipy.stats.linregress.html`.

(9) Virtanen, P. et al. SciPy 1.0: Fundamental Algorithms for Scientific Computing in Python. *Nature Methods* **2020**, *17*, 261–272.
